# Production of long-chain free fatty acids from metabolically engineered *Rhodobacter sphaeroides* heterologously producing periplasmic phospholipase A2 in dodecane-overlaid two-phase culture

**DOI:** 10.1186/s12934-019-1070-8

**Published:** 2019-01-31

**Authors:** Xiaomeng Tong, Eun Kyoung Oh, Byeong-ha Lee, Jeong K. Lee

**Affiliations:** Department of Life Science, Sogang University, Mapo, Shinsu 1, Seoul, 121-742 South Korea

**Keywords:** Long-chain free fatty acids, Phospholipase A2, Periplasmic expression, *Rhodobacter sphaeroides*, Photoheterotrophic conditions, High-cell-density culture, Dodecane-overlaid two-phase culture

## Abstract

**Background:**

Long-chain free fatty acids (FFAs) are a type of backbone molecule that can react with alcohol to produce biodiesels. Various microorganisms have become potent producers of FFAs. Efforts have focused on increasing metabolic flux to the synthesis of either neutral fat or fatty acyl intermediates attached to acyl carrier protein (ACP), which are the source of FFAs. Membrane lipids are also a source of FFAs. As an alternative way of producing FFAs, exogenous phospholipase may be used after heterologous production and localization in the periplasmic space. In this work, we examined whether *Rhodobacter sphaeroides*, which forms an intracytoplasmic membrane, can be used for long-chain FFA production using phospholipase.

**Results:**

The recombinant *R. sphaeroides* strain Rs-A2, which heterologously produces *Arabidopsis thaliana* phospholipase A2 (PLA2) in the periplasm, excretes FFAs during growth. FFA productivity under photoheterotrophic conditions is higher than that observed under aerobic or semiaerobic conditions. When the biosynthetic enzymes for FA (β-ketoacyl-ACP synthase, FabH) and phosphatidate (1-acyl-*sn*-glycerol-3-phosphate acyltransferase, PlsC) were overproduced in Rs-A2, the FFA productivity of the resulting strain Rs-HCA2 was elevated, and the FFAs produced mainly consisted of long-chain FAs of *cis*-vaccenate, stearate, and palmitate in an approximately equimolar ratio. The high-cell-density culture of Rs-HCA2 with DMSO in two-phase culture with dodecane resulted in an increase of overall carbon substrate consumption, which subsequently leads to a large increase in FFA productivity of up to 2.0 g L^−1^ day^−1^. Overexpression of the genes encoding phosphate acyltransferase (PlsX) and glycerol-3-phosphate acyltransferase (PlsY), which catalyze the biosynthetic steps immediately upstream from PlsC, in Rs-HCA2 generated Rs-HXYCA2, which grew faster than Rs-HCA2 and showed an FFA productivity of 2.8 g L^−1^ day^−1^ with an FFA titer of 8.5 g L^−1^.

**Conclusion:**

We showed that long-chain FFAs can be produced from metabolically engineered *R. sphaeroides* heterologously producing PLA2 in the periplasm. The FFA productivity was greatly increased by high-cell-density culture in two-phase culture with dodecane. This approach provides highly competitive productivity of long-chain FFAs by *R. sphaeroides* compared with other bacteria. This method may be applied to FFA production by other photosynthetic bacteria with similar differentiated membrane systems.

**Electronic supplementary material:**

The online version of this article (10.1186/s12934-019-1070-8) contains supplementary material, which is available to authorized users.

## Background

Given the increasing concern over the past decade regarding the projected depletion of fossil-fuel reserves and the concomitant emissions of greenhouse gases, the exploration of renewable and sustainable alternative biofuels is imperative. Much attention has been given lately to the microbial production of biofuels [1–3], including bioalcohol and biodiesel.

Recombinant *Escherichia coli* engineered for enhanced metabolic flow to ethanol provides significantly increased ethanol yield and productivity [4, 5]. Ethanol is both an important fuel blender and a starting resource for other basic raw materials [6]. However, in terms of energy density, ethanol is inferior to other biofuels with longer carbon chains [7].

Biodiesel is a monoalkyl ester derived from reactions between FFAs (usually longer than C10) and alcohols such as methanol, ethanol, propanol, and butanol. Biodiesel can be produced using edible oils as a source of FFAs, but the availability of edible feedstock in many countries may be low owing to the high demand for food resources [7]. Therefore, nonedible plant oils are used as alternative feedstocks; however, their supply requires large areas of cultivated land.

Given the need for higher productivity in limited space, microorganisms have been used as potent producers of FFAs and biodiesel [8]. Recombinant *E. coli* is able to produce alkanes, fatty alcohols, FFAs, and fatty esters of varying alkyl-chain lengths [8, 9]. *E. coli* has been further manipulated to achieve FFA productivity in the range of approximately 3–4.5 g L^−1^day^−1^ [10–12]. To enhance FFA production by *E. coli*, FFA uptake and degradation were blocked by the interruption of long-chain fatty acid transporter, FadL [13, 14] and acyl-CoA synthetase, FadD [15]. Moreover, FFA-biosynthesis genes were overexpressed with the simultaneous expression of either exogenous (plant) [15] or endogenous [13] thioesterase; the overexpression of genes encoding acetyl-CoA carboxylase (AccABCD) [16], β-ketoacyl-ACP synthase III (FabH) [17], or malonyl-CoA:ACP transacylase (FabD) [18] also increased FFA production by *E. coli*. FFA efflux in *E. coli* is mainly mediated by the AcrAB-TolC multidrug pump [19], which comprises TolC in the outer membrane, AcrB in the inner membrane and AcrA in the periplasmic space [20].

FFA production was also demonstrated in recombinant *Synechocystis* sp. PCC6803 [21]. The FFA secretion pathways common to recombinant strains of both *E. coli* and *Synechocystis* sp. PCC6803 consist mainly of two steps: FFA hydrolysis by thioesterase from fatty acyl-ACP inside the cell, followed by its export out of the cell. As an alternative method for the production of long-chain FFAs, a differentiated membrane can be used as a substrate for exogenous phospholipase in the periplasmic space. The secretion of FFAs from the periplasm could be facilitated more effectively than secretion from the cytoplasm because the outer membrane is the only export barrier.

The purple nonsulfur photosynthetic bacterium *Rhodobacter sphaeroides* gratuitously forms an intracytoplasmic membrane (ICM) in addition to the cell membrane when the partial pressure of oxygen (*p*O_2_) is lowered [22]. This phenomenon increases the cellular lipid content by approximately two- to threefold, resulting in levels similar to those of photoheterotrophically grown cells [23].

**Fig. 1:**
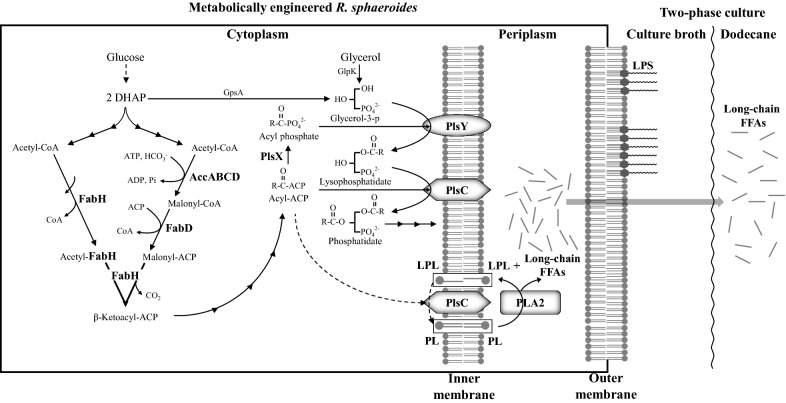
Production of FFAs from metabolically engineered *R. sphaeroides* in a two-phase culture system with dodecane. *R. sphaeroides* was recombinantly engineered to overproduce FabH, PlsX, PlsY, and PlsC in the cytoplasm and *A. thaliana* PLA2 in the periplasm. The long-chain FFAs released by *R. sphaeroides* were directed and localized to the dodecane layer. FFA sequestration in the dodecane layer alleviated the inhibitory effect of FFAs on cell growth, further elevating the FFA productivity of the cells. Multiple biosynthetic steps are illustrated by a series of connecting arrows, whereas a putative LPL acyltransferase activity of PlsC, which may form PL from LPL using acyl-ACP, is shown by dotted arrows. The PLs and LPLs of the inner membrane, which are the substrate and product of PLA2, respectively, are highlighted in boxes; the PLs of the outer membrane are also thought to be used by PLA2. DHAP, dihydroxyacetone phosphate; ACP, acyl carrier protein; FabH, β-ketoacyl-ACP synthase; AccABCD, acetyl-CoA carboxylase; FabD, malonyl-CoA:ACP transacylase; GpsA, glycerol-3-phosphate dehydrogenase; GlpK, glycerol kinase; PlsX, phosphate acyltransferase; PlsY, glycerol-3-phosphate acyltransferase; PlsC, 1-acyl-*sn*-glycerol-3-phosphate acyltransferase; LPS, lipopolysaccharide; LPL, lysophospholipid; PL, phospholipid; PLA2, phospholipase A2 of *A. thaliana*

In this study, we examined whether *R. sphaeroides* heterologously producing phospholipase A2 (PLA2) of *Arabidopsis thaliana*, which is subsequently localized in the periplasmic space (Fig. 1), can produce long-chain FFAs during photoheterotrophic growth. *R. sphaeroides* was metabolically engineered further to enhance metabolic flux to phospholipid (PL) formation by increasing the production of enzymes for the synthesis of FA and phosphatidate. Moreover, we tried high-cell-density culture to further increase FFA productivity. Because FFAs in the culture broth may be reutilized by cells, a two-phase culture system with dodecane (Fig. 1) was employed to keep the FFAs in the layer of organic solvent, preventing their reuse. Because FFAs are derived from cell membranes, long-chain (C_18_ and C_16_) FFAs are expected to be main components. In fact, the FFAs *cis*-vaccenate, stearate, and palmitate were obtained. This approach resulted in highly competitive productivity of long-chain FFAs compared with that observed in other photosynthetic bacteria [21, 24].

## Materials and methods

### Organisms and growth conditions

The wild-type (WT) strain *R. sphaeroides* KD131 [25] was cultured aerobically and photoheterotrophically at 28 °C in Sistrom’s succinate-based (Sis) minimal medium as described previously [25, 26]; Aerobic growth was performed by incubating 100 mL of culture in 1-L baffled flasks under vigorous shaking (250 rpm) on a gyratory shaker, while photoheterotrophic growth was obtained by incubating 100 mL of culture in completely filled vessels in the light (10 Watts/m^2^). Semiaerobic growth was achieved by incubating 100 mL of culture in 1-L baffled flasks under gentle agitation (50 rpm) on a gyratory shaker or by sparging (approximately 100 mL min^−1^ flow per 100 mL of culture) with a gas mixture of 2% O_2_, 1% CO_2_, and 97% N_2_. For high-cell-density culture, *R. sphaeroides* was grown in Sis minimal medium supplemented with succinate (up to 150 mM), glucose (100 mM), glycerol (150 mM), and yeast extracts (1% (w/v)) (SisH medium). When necessary, DMSO (10 mM) was added to the SisH medium (SisH-D medium). Dodecane (30%, v/v) was added to the bacterial broth (50–100 mL) for two-phase culture, which was mixed continuously with a magnetic stirring bar to ensure sufficient contact of dodecane with the cells. Cell aliquots were removed intermittently from the culture vessels for biochemical analyses; Cells grown photoheterotrophically were removed inside an anaerobic chamber (Model 10, COY, USA) to keep anaerobiosis, whereas cells grown aerobically and semiaerobically were removed under ambient conditions. *Escherichia coli* was grown at 37 °C in Luria–Bertani medium. Antibiotics for *R. sphaeroides* and *E. coli* cultures were used at concentrations previously indicated [27]. *A. thaliana* was grown photosynthetically under a light intensity of 110 ± 10 μmol m^−2^s^−1^ and a 16-h/8-h light–dark cycle at 22 ± 1 °C with 60 ± 10% humidity, as described previously [28].

### Preparation of DNA fragments encoding the enzymes for the metabolism of FAs and PLs

All DNA fragments containing genes encoding enzymes involved in the metabolism of FAs and PLs were PCR-amplified from *R. sphaeroides* genomic DNA, whereas the genes for phospholipases [phospholipase A1 (PLA1) and phospholipase A2 (PLA2)] were obtained from *A. thaliana* cDNA. The cDNA sequence of *A. thaliana* was obtained from http://www.arabidopsis.org/index.jsp, and cDNA was synthesized as follows. Total RNA was extracted from 2-week-old *Arabidopsis* plants using a Plant RNA Kit (Nanohelix, Daejeon, Korea). DNase I (New England Biolabs, Ipswich, MA, USA) was added to the total RNA to remove genomic DNA. Using the total RNA as templates, first-strand cDNA was synthesized by PrimeScript reverse transcriptase (Takara Bio, Shiga, Japan) according to the manufacturer’s protocol. Briefly, total RNA (5 μg) was mixed with oligo dT primer (5 μM) and dNTP (2 mM) in 10 μL of H_2_O and incubated at 65 °C for 5 min, followed by mixing with 20 U RNase inhibitor and 100 U reverse transcriptase in PrimeScript buffer (Takara). The reaction proceeded at 42 °C for 60 min, and the reverse transcriptase was heat-inactivated at 70 °C for 5 min to stop the reaction.

### Construction of plasmids and recombinant strains

#### pINDA′-PLA1, pIND-PLA2 and pINDA′-PLA2

The 2.0-kb *Sma*I/*Stu*I transcription and translation stop Ω [streptomycin/spectinomycin-resistant (Sm^r^/Sp^r^)] DNA was inserted into the *Sma*I site of the kanamycin-resistant (Km^r^) gene of pIND4 [29] to obtain pIND4S, a recombinant plasmid that could then be maintained in a cell using Sm/Sp compatibly with the plasmid derived from pBBR1MCS-2 (Km^r^) (Table 1). Among the fourteen phospholipases A1 (PLA1) of *A. thaliana*, At1g06800.1 was chosen for further cloning due to its broad specificity for PL head groups [30, 31]. To construct a pIND4S-derived plasmid carrying an insert DNA encoding PLA1 fused to the CycA signal peptide sequence (CycA′) of *R. sphaeroides*, the 63-bp *Nco*I–*Xba*I fragment encoding CycA′ was PCR-amplified from *R. sphaeroides* genomic DNA using primers CycA-F and CycA-R (Additional file 1: Table S1). Then, a 1.5-kb *Xba*I–*Hin*dIII fragment encoding PLA1, extending from its start codon to the termination codon, was PCR-amplified from *A. thaliana* cDNA using primers PLA1-F and PLA1-R (Additional file 1: Table S1). The two DNA fragments were digested with *Nco*I–*Xba*I and *Xba*I–*Hin*dIII, respectively, and cloned into the *Nco*I/*Hin*dIII sites of pIND4S to generate pINDA′-PLA1 (Table 1).

**Table 1:** Strains and plasmids used in this study

Strains/plasmids	Relevant characteristics	References/description
Strains		
*E. coli*		
DH5α*phe*	*phe*::Tn10*d*Cm of DH5α	[32]
HB101	*F*^−^ *mcrB mrr hsdS20(r*_*B*_^−^ *m*_*B*_^−^*) recA13 leuB6 ara*-*14 proA2 lacY1 galK2 xyl*-*5 mtl*-*1 rpsL20(Sm*^*R*^*) glnV44 λ*^−^	[33]
S17-1	C600::RP4 2-(Tc::Mu)(Km::Tn7) *thi pro hsdR hsdM*^+^ *recA*	[34]
*R. sphaeroides*		
KD131	Type strain	[25]
Rs-nA2	*R. sphaeroides* containing pIND-PLA2	This study
Rs-A2	*R. sphaeroides* containing pINDA′-PLA2	This study
Rs-nA2-phoA	*R. sphaeroides* containing pIND-PLA2-phoA	This study
Rs-A2-phoA	*R. sphaeroides* containing pINDA′-PLA2-phoA	This study
Rs-C	*R. sphaeroides* containing pLVplsC	This study
Rs-DC	*R. sphaeroides* containing pRKfabD and pLVplsC	This study
Rs-AccC	*R. sphaeroides* containing pRKaccABCD and pLVplsC	This study
Rs-HC	*R. sphaeroides* containing pRKfabH and pLVplsC	This study
Rs-AccCA2	*R. sphaeroides* containing pRKaccABCD, pLVplsC and pINDA′-PLA2	This study
Rs-CA2	*R. sphaeroides* containing pLVplsC and pINDA′-PLA2	This study
Rs-DCA2	*R. sphaeroides* containing pRKfabD, pLVplsC and pINDA′-PLA2	This study
Rs-HCA2	*R. sphaeroides* containing pRKfabH, pLVplsC and pINDA′-PLA2	This study
Rs-HXYCA2	*R. sphaeroides* containing pRKfabH, pBBRplsX, pLVplsCY and pINDA′-PLA2	This study
Rs-tol	A *tolC* mutant; Tp^r^	This study
Rs-A2tol	A *tolC* mutant containing pINDA′-PLA2; Tp^r^	This study
Plasmids		
pBBR1MCS-2	*ori* pBBR1 *lac*Za; Km^r^	[35]
pBBRplsX	pBBR + 1.3-kb *Hin*dIII–XbaI fragment containing *PlsX*; Km^r^	This study
pBS(−)	*ori* f1 *lac*Z′; Ap^r^	Stratagene
pBSaccA	pBS + 1.1-kb *Xba*I–*Pst*I fragment containing *accA*; Ap^r^	This study
pBSaccD	pBS + 1.3-kb *Xba*I–*Pst*I fragment containing *accD*; Ap^r^	This study
pBSfabD	pBS + 1.2-kb *Xba*I–*Pst*I fragment containing *fabD*; Ap^r^	This study
pMD20	*ori* f1 *lac*Z′; Ap^r^	Takara
pMDaccBC	pMD + 2.9-kb *Hin*dIII–*Xba*I fragment containing *accBC*; Ap^r^	This study
pIND4	*ori* ColE1 IPTG-inducible promoter; Km^r^	[29]
pIND4S	*ori* ColE1 IPTG-inducible promoter; Sm/Sp^r^	This study
pIND-PLA2	pIND4S + 387-bp *Nco*I–*Hin*dIII fragment containing *PLA2*; Sm/Sp^r^	This study
pINDA′-PLA2	pIND4S + 456-bp *Nco*I–*Hin*dIII fragment containing *cycA′*-*PLA2*; Sm/Sp^r^	This study
pINDA′-PLA1	pIND4S + 1.9-kb *Nco*I–*Hin*dIII fragment containing *cycA′*-*PLA1*; Sm/Sp^r^	This study
pIND-PLA2-phoA	pIND4S + 1.9-kb *Nco*I–*Hin*dIII fragment containing *PLA2*-*phoA*; Sm/Sp^r^	This study
pINDA′-PLA2-phoA	pIND4S + 2.0-kb *Nco*I–*Hin*dIII fragment containing *cycA′*-*PLA2*-*phoA*; Sm/Sp^r^	This study
pLV106	IncQ pUI511 containing the polylinker of *Eco*RI, *Sma*I, *Kpn*I, and *Xba*I restriction sites inserted at *Pst*I site	[36]
pLV-Tp	pLV106 + 1.6-kb *Hin*dIII fragment containing Tp^r^ gene	This study
pLVplsC	pLV-Tp + 1.0-kb *Xba*I–*Kpn*I fragment containing *pls*C; Tp^r^	This study
pLVplsCY	pLV-Tp + 1.0-kb *Xba*I–*Kpn*I fragment containing *pls*C and 0.8-kb *Kpn*I-*Eco*RI fragment containing *pls*Y; Tp^r^	This study
pRK2013	*ori* ColE1; Km^r^	[37]
pRK415	*ori* IncP-1 Mob RP4 *lac*Za; Tc^r^	[38]
pRKfabH	pRK415 + 1.8-kb *Kpn*I–*Xba*I fragment containing *fab*H; Tc^r^	This study
pRKfabD	pRK415 + 1.2-kb *Xba*I–*Kpn*I fragment containing *fab*D; Tc^r^	This study
pRKaccABCD	pRK415 + 5.3-kb *Xba*I–*Kpn*I fragment containing *accABCD*; Tc^r^	This study
pSUP202	*ori* p15A Mob RP4; Tc^r^ Cm^r^ Ap^r^	[39]
pSUPTolC	pSUP202 + 2.6-kb DNA of *tolC*::Tp; *tolC* interrupted by 1.6-kb Tp^r^ DNA; Tp^r^ Tc^r^	This study

Among the four instances of phospholipase A2 (PLA2) in *A. thaliana*, At2g06925 was chosen for further cloning due to its broad optimal pH range and relatively weak preference for PL head groups [40]. To construct a pIND4S-derived plasmid carrying an insert DNA encoding PLA2, a 387-bp *Nco*I–*Hin*dIII fragment encoding PLA2 without its signal peptide of twenty N-terminal residues was PCR-amplified from *A. thaliana* cDNA using primers PLA2-F and PLA2-R (Additional file 1: Table S1). The DNA fragment was digested with *Nco*I/*Hin*dIII and cloned into the *Nco*I/*Hin*dIII sites of pIND4S to generate pIND-PLA2 (Table 1), in which signal peptide-less PLA2 was oriented in-frame to the start codon at the multiple cloning sites of the plasmid.

To construct a pIND4S-derived plasmid carrying an insert DNA encoding PLA2 fused to CycA′ of *R. sphaeroides*, the 63-bp *Nco*I–*Xba*I fragment encoding CycA′ was PCR-amplified from *R. sphaeroides* genomic DNA as described above, and a 387-bp *Xba*I–*Hin*dIII fragment encoding PLA2 without its signal peptide was PCR-amplified from *A. thaliana* cDNA, using primers PLA2-F´ and PLA2-R (Additional file 1: Table S1). The two DNA fragments encoding CycA′ and signal peptide-less PLA2 were digested with *Nco*I–*Xba*I and *Xba*I–*Hin*dIII, respectively, and cloned into the *Nco*I/*Hin*dIII sites of pIND4S to generate pINDA′-PLA2 (Table 1).

#### pIND-PLA2-phoA and pINDA′-PLA2-phoA

A 1.5-kb *Pst*I–*Hin*dIII fragment encoding alkaline phosphatase (PhoA) without its thirty-three N-terminal residues (signal peptide-less PhoA (PhoA′)) was PCR-amplified from *E. coli* genomic DNA using primers PhoA-F and PhoA-R (Additional file 1: Table S1) and then cloned into the *Pst*I/*Hin*dIII sites of pBS(−) (Stratagene, San Diego, CA, USA) to generate pBSPhoA. A 384-bp *Nco*I–*Pst*I fragment encoding PLA2, extending from the 21st codon of PLA2 to the second from the termination codon, was PCR-amplified from pIND-PLA2 using primers PLA2-t-F and PLA2-t-R (Additional file 1: Table S1). Likewise, a 453-bp *Nco*I–*Pst*I fragment encoding CycA′-PLA2, extending from the start codon of CycA′ to the second from the termination codon of PLA2, was PCR-amplified from pINDA′-PLA2 using primers CycA-F and PLA2-t-R (Additional file 1: Table S1). These two DNA fragments were digested with *Nco*I–*Pst*I, and each was cloned with a 1.5-kb *Pst*I–*Hin*dIII fragment of PhoA′ of pBSPhoA into the *Nco*I/*Hin*dIII sites of pIND4S to yield pIND-PLA2-phoA and pINDA′-PLA2-phoA, respectively (Table 1).

#### pRKaccABCD

A 1.1-kb *Xba*I–*Pst*I fragment extending from 171 bp upstream of the start codon of AccA to its termination codon was PCR-amplified using primers AccA-F and AccA-R (Additional file 1: Table S1). The resulting DNA fragment was digested with *Xba*I and *Pst*I and ligated into the *Xba*I/*Pst*I sites of pBS(−) (Stratagene) to generate pBSaccA (Table 1). Similarly, a 1.3-kb *Xba*I-*Pst*I fragment extending from 363 bp upstream of AccD to its termination codon was PCR-amplified using primers AccD-F and AccD-R (Additional file 1: Table S1). The resulting DNA fragment was digested with *Xba*I and *Pst*I and ligated into the *Xba*I/*Pst*I sites of pBS(−) to yield pBSaccD (Table 1). The 2.9-kb *Hin*dIII/*Xba*I *accBC* DNA extending from 712 bp upstream of the AccB start codon to 233 bp downstream of AccC was PCR-amplified using primers AccBC-F and AccBC-R (Additional file 1: Table S1). The resulting fragment was ligated into T-vector pMD20 (Takara) to generate pMD-accBC (Table 1). The 1.1-kb *Xba*I–*Hin*dIII fragment containing *accA*, the 2.9-kb *Hin*dIII–*Xba*I fragment containing *accBC*, and the 1.3-kb *Xba*I–*Kpn*I fragment containing *accD* were digested from pBSaccA, pMDaccBC, and pBSaccD, respectively, with restriction enzymes, followed by sequential ligation into the *Xba*I/*Kpn*I sites of pRK415 to generate pRKaccABCD (Table 1).

#### pRKfabD

A 1.2-kb *Xba*I–*Pst*I DNA fragment extending from 228 bp upstream of the FabD start codon to its termination codon was PCR-amplified using primers FabD-F and FabD-R (Additional file 1: Table S1). The resulting DNA fragment was digested with *Xba*I and *Pst*I and ligated into the *Xba*I/*Pst*I sites of pBS(−) to generate pBSfabD (Table 1). The 1.2-kb *Xba*I–*Kpn*I DNA containing *fabD* was digested from pBSfabD by restriction enzymes and cloned into the *Xba*I/*Kpn*I sites of pRK415 to generate pRKfabD (Table 1).

#### pRKfabH

A 1.8-kb *Kpn*I–*Xba*I fragment extending from 586 bp upstream of the FabH start codon to 194 bp downstream of the termination codon was PCR-amplified using primers FabH-F and FabH-R (Additional file 1: Table S1). The resulting DNA fragment was digested with *Kpn*I and *Xba*I and ligated into the *Kpn*I/*Xba*I sites of pRK415 to generate pRKfabH (Table 1).

#### pLVplsC and pLVplsCY

A 970-bp *Xba*I–*Kpn*I fragment extending from 177 bp upstream of the PlsC start codon to 78 bp downstream of the termination codon was PCR-amplified using primers PlsC-F and PlsC-R (Additional file 1: Table S1). The resulting DNA fragment was digested with *Xba*I and *Kpn*I and ligated into the *Xba*I/*Kpn*I sites of pLV106 to generate pLVplsC (Table 1). A 0.8-kb *Kpn*I-*Eco*RI fragment extending from 220 bp upstream of the PlsY start codon to 21 bp downstream of the termination codon was PCR-amplified using primers PlsY-F and PlsY-R (Additional file 1: Table S1). The resulting DNA fragment was digested with *Kpn*I and *Eco*RI and ligated into the *Kpn*I/*Eco*RI sites of pLVplsC to generate pLVplsCY (Table 1), in which *plsC* and *plsY* are oriented in the same direction.

#### pBBRplsX

A 1.3-kb *Hin*dIII–*Xba*I DNA fragment extending from 167 bp upstream of the PlsX start codon to 42 bp downstream from the termination codon was PCR-amplified using primers PlsX-F and PlsX-R (Additional file 1: Table S1). The resulting DNA fragment was digested with *Hin*dIII and *Xba*I and ligated into the *Hin*dIII*/Xba*I sites of pBBR1MCS-2 to generate pBBRplsX (Table 1).

#### Construction of the *R. sphaeroides* tolC mutant Rs-tol

A 550-bp *Pst*I–*Bam*HI fragment upstream of the codon encoding the 2nd residue of TolC was PCR-amplified from *R. sphaeroides* genomic DNA using primers TolC-UF and TolC-UR (Additional file 1: Table S1). A 549-bp *Hin*dIII-*Eco*RI fragment downstream of the 465th residue of TolC was PCR-amplified using primers TolC-DF and TolC-DR (Additional file 1: Table S1). In parallel, a 1.6-kb *Bam*HI/*Hin*dIII fragment containing the trimethoprim (Tp)-resistance (Tp^r^) gene was obtained from pLV-Tp (Table 1). The three fragments, including the 550-bp *Pst*I–*Bam*HI fragment, the 1.6-kb *Bam*HI–*Hin*dIII Tp^r^ gene, and the 549-bp *Hin*dIII–*Eco*RI fragment, were ligated sequentially into the *Pst*I/*Eco*RI sites of pSUP202 [39] to generate pSUPtolC (Table 1), from which the *tolC* DNA encoding the polypeptide between the 2nd and 465th residue was deleted. pSUPtolC was mobilized into *R. sphaeroides*, and a Tp^r^ and Tc^s^ double-crossover recombinant was isolated as previously described [41]. The chromosome structure of the Rs-tol mutant (Table 1) was confirmed by Southern hybridization analysis.

### Mobilization of recombinant plasmids into *R. sphaeroides*

Recombinant plasmids were mobilized into *R. sphaeroides* using *E. coli* S17-1 (Table 1), as described previously [41]. For plasmids carrying the Tp^r^ gene, *E. coli* DH5α*phe* (Table 1) was used for conjugation [42].

### Alkaline phosphatase (PhoA) assay

PhoA activity was detected using 5-bromo-4-chloro-3-indolyl phosphate/nitro blue tetrazolium (BCIP/NBT) solution (Sigma-Aldrich, St. Louis, MO, USA), as described previously [43]. Aliquots of the BCIP/NBT solution (one tablet in 3 mL of H_2_O) were dropped onto colonies on Sis agar plates and incubated at 30 °C for 5 min.

### Preparation of cytoplasmic and periplasmic fractions

The cytoplasmic and periplasmic fractions of *R. sphaeroides* were prepared as described previously [44]. Cells were grown under photoheterotrophic conditions and harvested at the exponential growth phase by centrifugation at 3000*g* and 4 °C for 20 min. The cell pellet was gently suspended in 1 mL of Tris-sucrose-EDTA (200 mM Tris–HCl, pH 8.0, 500 mM sucrose, 1 mM EDTA) buffer and incubated on ice for 30 min, followed by centrifugation at 16,000*g* and 4 °C for 30 min. The supernatant was recovered, representing the periplasmic fraction. The pellet containing spheroplasts was suspended in 20 mM sodium borate buffer (pH 9.0) and sonicated on ice, then centrifuged at 12,000*g* and 4 °C for 10 min. The resulting supernatant represented the cytoplasmic fraction.

### Phospholipase assay

The phospholipase assay was carried out as described previously [45]. Membrane PLs were extracted from photoheterotrophically grown *R. sphaeroides* [46] and used as substrates for the assay. The reaction mixture contained an aliquot (35 µg protein) of either the cytoplasmic or periplasmic fraction (“Preparation of cytoplasmic and periplasmic fractions” section) and 400 µg of PLs in 1 mL of 20 mM sodium borate buffer (pH 9.0) containing 1 mM CaCl_2_. The reaction was performed at 30 °C for 15 min, and terminated by heating at 100 °C for 5 min. The reaction mixture was then centrifuged at 12,000*g* and 4 °C for 10 min, and the supernatant (50 µL) was examined for FFA using the FFA Quantification Colorimetric/Fluorometric Kit (BioVision, Milpitas, CA, USA). The reaction was performed with no cell fraction aliquot as a control, and a standard curve was prepared with varying concentrations of palmitate. One enzyme unit was defined as 1 µmol of FFAs released per min from PLs.

### RNA isolation and quantitative reverse transcription PCR (RT-qPCR)

Cells were harvested during exponential growth, and total RNA was isolated using the RNeasy Mini Kit (Qiagen, Valencia, CA) according to the manufacturer’s protocol. RNase-free DNase (Qiagen) was used to remove DNA. The PrimeScript RT Reagent Kit (Takara Bio, Shiga, Japan) was used to reverse transcribe RNA using primers specific to each gene (Additional file 1: Table S1), and the cDNA obtained from each sample (1 μg) was used for further analysis. Relative mRNA quantification was performed using the LightCycler 480 real-time PCR system (Roche, Basel, Switzerland) with the SYBR Premix Ex Taq (Tli RNaseH Plus) Kit (Takara Bio). The amplification efficiencies of interest and reference genes were equal to 100%. Final mRNA levels were normalized to that of the housekeeping 16S rRNA by the 2^−ΔΔCT^ method [47]. All the quantifications were independently repeated three times, and data are shown as the mean ± standard deviation (SD).

### Thin-layer chromatography

Total lipids were extracted from cells as described previously [48]. The extracted lipids were dissolved in chloroform and loaded onto activated silica thin-layer chromatography (TLC) plates (Z185329, Sigma-Aldrich). The plate was developed with a solvent mixture of chloroform–methanol–acetic acid–water (85:15:10:3.5, v/v) [48], then air-dried and exposed to iodine vapor for approximately 20 min at room temperature until the bands appeared. To determine the lysophospholipids (LPLs) in the cell membrane, a mixture of chloroform–methanol–acetic acid–acetone–water (35:25:4:14:2, v/v) [49] was used as the development solvent instead.

### Spectral complex determination

Cells were grown under photoheterotrophic conditions and harvested at the exponential growth phase. Cell-free lysates were prepared and analyzed for spectral complexes as described previously [22, 50].

### Malate dehydrogenase assay

Cells were harvested and cell-free lysates were prepared as described in “Spectral complex determination” section. The culture supernatant was concentrated approximately tenfold by centrifugation with Amicon^®^ Ultra4 centrifugal Filters-3K (Merck, Ireland). The enzyme reaction was performed at 25 °C by mixing 12 μg of protein, from either concentrated culture supernatant or cell-free extracts, with substrates (NADH and oxaloacetate at final concentrations of 0.5 mM and 0.2 mM, respectively) in 0.1 M potassium phosphate buffer (pH 7.4) at a final volume of 0.5 mL. Malate dehydrogenase activity was determined by measuring the decrease in absorbance of NADH at 340 nm, as described previously [51].

### Determination of cellular FAs (CFAs) and FFAs in culture broth

CFAs of *R. sphaeroides* were determined as described previously [46]. Standard fatty acid methyl esters (FAMEs) (37-component FAME; Sigma-Aldrich) were used to determine the retention times of sample FAMEs in gas-chromatographic analysis. *cis*-vaccenic acid (Sigma-Aldrich) was esterified for use as a control because it is not included as a standard FAME. An aliquot of pentadecanoic acid (Sigma-Aldrich) was used as an internal standard. To determine the FFAs in culture broth, 3 mL of culture supernatant was mixed with 10 µg of pentadecanoic acid, followed by solvent extraction with chloroform and methanol (2:1 [v/v]) for 2 h with shaking at room temperature. The chloroform layer was evaporated under a nitrogen stream, and the FFAs were determined as described previously [46]. When cells were cultured in two-phase system with dodecane, an aliquot of the solvent layer was removed and centrifuged to obtain 100 µL of dodecane, which was analyzed for FFAs in the same way as the culture supernatant sample.

FFA productivity was determined from the FFA level in culture supernatant (and in the dodecane in dodecane-overlaid culture) harvested at several time points from exponential phase to the beginning part of the stationary phase. The FFA productivity was derived from the FFA levels at a given sampling point divided by the time at that sampling point, which had elapsed after inoculation. The maximal FFA productivities at exponential growth phase, which remained unchanged to the beginning part of the stationary phase, were regarded as a representative FFA productivity. In contrast, FFA titer was determined from the maximal level of FFAs in culture supernatant (and in the dodecane in dodecane-overlaid culture) during the stationary phase.

### Preparation of potassium *cis*-vaccenate

One gram of *cis*-vaccenic acid (Sigma-Aldrich, USA) was dissolved in 10 mL of alcoholic KOH (40 g L^−1^), followed by boiling for 1 h. The solution was cooled to room temperature, neutralized with 0.5 N HCl, and then completely dried at 65 °C and used as potassium *cis*-vaccenate.

### Determination of glucose, glycerol and succinate

Aliquots of cell culture were centrifuged to obtain the supernatant, which was stored at 4 °C for further analysis. The glucose concentration was examined using a Glucose Assay Kit (Sigma-Aldrich). A Free Glycerol Colorimetric/Fluorometric Assay Kit (BioVision, Milpitas, CA, USA) was used to determine glycerol levels, and a Succinate (Succinic Acid) Colorimetric Assay Kit (BioVision) was used to examine the succinate levels in the culture supernatant.

## Results and discussion

### FFAs are released from the photoheterotrophically grown recombinant *R. sphaeroides* (Rs-A2) heterologously producing *Arabidopsis thaliana* PLA2, which is localized in the periplasm

The CFA content of *R. sphaeroides* KD131 grown under photoheterotrophic and semiaerobic conditions is approximately twofold higher than that of cells grown aerobically (Additional file 1: Fig. S1), which is attributed to the formation of ICM under O_2_-limited conditions [22]. Cells grow photoheterotrophically with a doubling time half that observed under semiaerobic conditions (Additional file 1: Fig. S1), and the resulting maximal turbidity is nearly doubled in the same comparison (Additional file 1: Fig. S1). In this work, we heterologously produced *A. thaliana* phospholipases, which are genetically engineered to be localized to the periplasmic space, in *R. sphaeroides*, and then addressed the question of whether the recombinant strain can produce FFAs (Fig. 1). Given that the cell membrane is a barrier to FFA translocation, the export of FFAs from the periplasm may be easier than secretion from the cytoplasm.

PLA1 and PLA2 of *A. thaliana* have been biochemically characterized in detail [31, 40] and used in this study. PLA1 of *A. thaliana* was fused in-frame to the signal peptide sequence (CycA′) of the periplasmic protein cytochrome *c*_2_ (CycA) of *R. sphaeroides* to generate the plasmid pINDA′-PLA1 (Table 1). When pINDA′-PLA1 was mobilized into WT cells, we failed to obtain any stable exconjugant, even from several independent trials. The reaction product of PLA1 is 2-acyl LPL, and its accumulation in the membrane might be toxic to *R. sphaeroides*. In contrast, pINDA′-PLA2 (Table 1), in which signal peptide-less PLA2 from *A. thaliana* was fused in-frame to CycA′, was successfully mobilized into *R. sphaeroides* to yield the exconjugant strain Rs-A2 (Table 1), which was used in the subsequent experiments. The cloning of signal peptide-less PLA2 without CycA′ generated pIND-PLA2 (Table 1), and its exconjugant Rs-nA2 (Table 1) was used as a control.

**Fig. 2:**
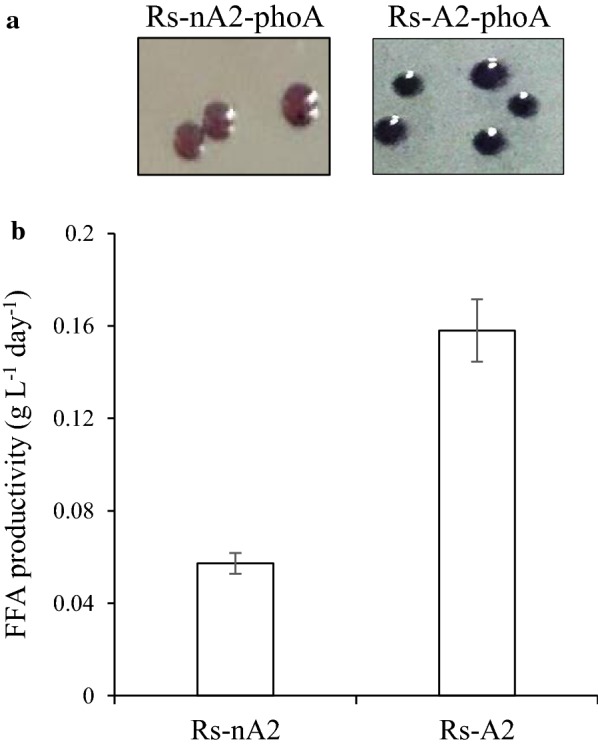
Heterologous production of PLA2 in the periplasm of Rs-A2 and its FFA productivity. **a** PhoA activity of Rs-A2-phoA was examined with BCIP/NBT solution, which was dropped onto colonies grown aerobically on Sis minimal medium agar plates and incubated at 30 °C for 5 min (right). Rs-nA2-phoA colonies were used as a control (left). **b** *R. sphaeroides* heterologously producing PLA2 in either the cytoplasm (Rs-nA2) or periplasm (Rs-A2) was grown photoheterotrophically and harvested at the exponential growth phase (OD_660_ of 2.3). The FFA level in the culture supernatant was measured, and the FFA productivity is shown with error bars from three independent experiments

Rs-A2 was grown aerobically, semiaerobically and photoheterotrophically and harvested intermittently at several time points extending from exponential to stationary growth phases (Additional file 1: Fig. S2, A1, B1, and C1). FFA productivity usually reaches the maximal level at the end of exponential growth and remained unchanged to the beginning part of stationary phase under aerobic and photoheterotrophic conditions (Additional file 1: Fig. S2, A1 and C1), whereas the FFA productivity at low O_2_ reaches the maximal level at the exponential growth phase and gradually decreased during stationary phase (Additional file 1: Fig. S2, B1). The maximal FFA productivities at the exponential growth phase was regarded as the representative value of the recombinant strain. On the other hand, FFA titer usually reaches the maximal level during the stationary phase (Additional file 1: Fig. S2, A1, B1 and C1). Thus, Rs-A2 showed an FFA productivity of approximately 0.15 ~ 0.16 g L^−1^ day^−1^ under photoheterotrophic conditions (Fig. 2b, Additional file 1: Fig. S2, C1 and Fig. S3), which was approximately twofold higher than that of the aerobically grown cells (Additional file 1: Fig. S2, A1 and Fig. S3). Semiaerobically grown cells showed intermediate FFA productivity between those of the cells grown aerobically and photoheterotrophically (Additional file 1: Fig. S2, B1 and Fig. S3). Thus, cells were grown photoheterotrophically for higher FFA productivity in the subsequent experiments, unless specified otherwise.

FFA efflux in *E. coli* is mainly mediated by the AcrAB-TolC multidrug pump [19], which comprises TolC in the outer membrane and AcrB and AcrA in the inner membrane and periplasmic space [20], respectively. Likewise, *Salmonella enterica* serovar Typhimurium employs TolC, which has 94% similarity (90% identity) to *E. coli* TolC, to excrete long-chain FFAs into the culture medium [52]. We investigated whether FFAs from Rs-A2 utilized a similar export system. The *R. sphaeroides* TolC homolog (Rsp_1576), which shows approximately 41–43% similarity (23–24% identity) to the TolCs of *E. coli* and *Salmonella*, was interrupted to yield the Rs-tol strain (Table 1). Plasmid pINDA′-PLA2 was then mobilized into Rs-tol to yield the exconjugant Rs-A2tol (Table 1), and its FFA productivity was compared with that of Rs-A2. There was no difference in FFA productivity between Rs-A2 and its isogenic *tolC*-interruption mutant (Additional file 1: Fig. S4). Accordingly, our results suggest that FFAs from Rs-A2 are exported from the periplasmic space through a TolC-independent pathway.

**Fig. 3:**
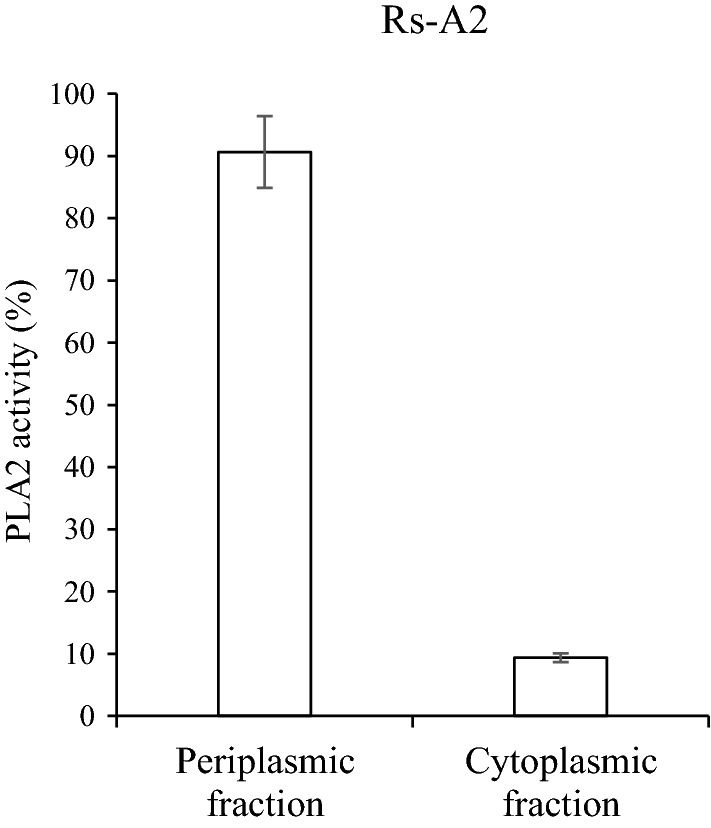
PLA2 activities of the periplasmic and cytoplasmic fractions of Rs-A2. Rs-A2 was grown photoheterotrophically in Sis minimal medium. Periplasmic and cytoplasmic fractions were prepared from cells harvested at the exponential growth phase (OD_660_ of 2.3). The PLA activity in each fraction was determined with membrane lipids from the photoheterotrophically grown WT cells and expressed as the percentage of the total activity, which amounted to approximately 111.5 U mg^−1^ protein. One enzyme unit was defined as 1 µmol of FFAs released per min from PLs under the assay conditions examined. PLA2 activities are shown with error bars from three independent experiments

**Fig. 4:**
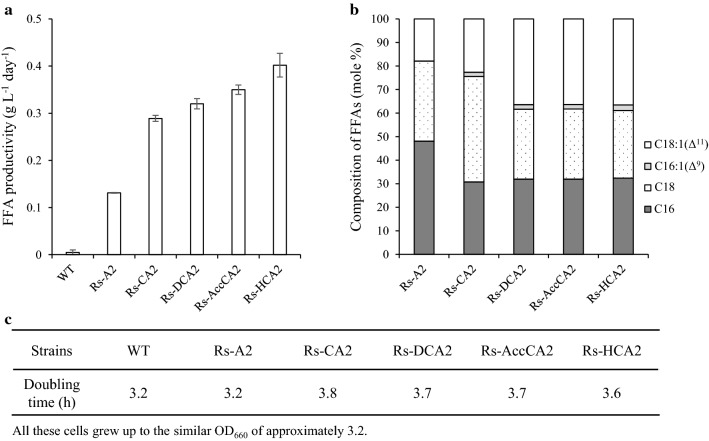
FFA productivity, FFA composition, and growth of Rs-A2, Rs-CA2, Rs-DCA2, Rs-AccCA2, and Rs-HCA2. **a** The recombinant *R. sphaeroides* strains were grown photoheterotrophically in Sis minimal medium and harvested at the exponential growth phase (OD_660_ of 2.5, 2.3, 2.3, 2.4, 2.3 and 2.2 for WT, Rs-A2, Rs-CA2, Rs-DCA2, Rs-AccCA2 and Rs-HCA2, respectively). FFAs in the culture supernatant were determined, and the FFA productivity is shown with error bars from three independent experiments. **b** The composition of FFAs is illustrated as the percentage of the total moles of FFAs. The experiments were independently repeated three times; the data shown are for one of three representative experiments. **c** Doubling times of recombinant *R. sphaeroides* strains are shown with WT cells as a control. The data presented here were reproduced within SDs of 10–15%. The experiments were independently repeated three times, and the data shown are for one of three representative experiments

To determine the cellular location of PLA2, PhoA′ was fused in-frame to the PLA2 of pINDA′-PLA2, followed by mobilization into WT cells to generate Rs-A2-phoA (Table 1). Likewise, pIND-PLA2-phoA was constructed and mobilized into WT cells to generate Rs-nA2-phoA (Table 1), in which PLA2 was produced without the signal peptide, CycA′. Activity staining of Rs-A2-phoA colonies with BCIP/NBT solution revealed blue staining, indicating PhoA activity in the periplasmic space, whereas colonies of Rs-nA2-PhoA showed no blue staining (Fig. 2a). Consistently, Rs-nA2 showed FFA productivity one-third that of Rs-A2 (Fig. 2b). Approximately 90% of the phospholipase activity was localized to the periplasmic fraction of Rs-A2, and the remaining 10% of phospholipase activity was found in the cytoplasmic fraction (Fig. 3). Thus, the results indicate that PLA2 of Rs-A2 is heterologously produced and localized in the periplasmic space. Heterologous production of PLA2 did not affect the photoheterotrophic growth of Rs-A2, as shown by the doubling time and maximal cell growth, which are similar to the corresponding values of WT cells (Fig. 4c).

Monounsaturated FA is the best component for biodiesel because it provides low-temperature fluidity and appropriate oxidative stability [53, 54]. *R. sphaeroides* harbors CFAs consisting of approximately 70% *cis*-vaccenic acid [55], and unsaturated FA is expected to be found most frequently at the *sn*2 carbon of PL. Analysis of the FFAs in the culture supernatant of Rs-A2, however, revealed approximately 20% *cis*-vaccenate at most (Fig. 4b). The remaining 80% of the FFAs mainly consist of stearate (~ 35%) and palmitate (~ 45%). The composition of FFAs did not vary during cell growth (Additional file 1: Fig. S2, A2, B2 and C2). Likewise, the heterologous expression levels of *pla2* remained unchanged during the growth (Additional file 1: Fig. S2, A3, B3 and C3). The lower-than-expected *cis*-vaccenic acid content among the FFAs suggests its importance in the membrane for the viability and growth of *R. sphaeroides*.

### Overproduction of FabH and PlsC in Rs-A2, generating Rs-HCA2, results in approximately a threefold increase in FFA productivity

We then examined whether the FFA productivity can be further increased by overproducing enzymes that catalyze the metabolic steps to form phosphatidate, a precursor of PL (Fig. 1). Because PLA2 hydrolyzes FAs from the *sn*2 carbon of PLs, the resulting LPL may need to be converted into PL again by LPL acyltransferase. However, no ORF of the *R. sphaeroides* genome has been specifically annotated as LPL acyltransferase. The 1-acyl-*sn*-glycerol-3-phosphate acyltransferase PlsC is known to exhibit LPL acyltransferase activity [56, 57]. Rsp_0735 has approximately 50% amino acid sequence identity with PlsC of *Rhodobacter capsulatus* (Additional file 1: Fig. S5), which was shown to complement the *plsC* mutant of *E. coli* [58]. When the gene encoding putative PlsC (Rsp_0735) was overexpressed in Rs-A2, the FFA productivity of the resulting recombinant strain Rs-CA2 (Table 1) was increased approximately twofold (Fig. 4a). We examined whether LPLs are present in the membranes of Rs-A2 and Rs-CA2 (Additional file 1: Fig. S6). Some LPLs, including lysophosphatidylethanolamine (lysoPE), were found to be associated with the ICM of *R. capsulatus* [59]. Interestingly, there is no difference in the PL and lysoPE profiles of the membranes of WT, Rs-A2 and Rs-CA2 cells (Additional file 1: Fig. S6), suggesting the rapid conversion of LPLs into PLs in these recombinant cells.

We further overexpressed the genes encoding enzymes that catalyze either the formation of malonyl-ACP (malonyl-CoA:ACP transacylase, FabD; acetyl-CoA carboxylase, AccABCD) or the initial condensation to form acetoacetyl-ACP (β-ketoacyl-ACP synthase, FabH) in Rs-CA2. FFA productivities of the resulting recombinant strains Rs-DCA2, Rs-AccCA2, and Rs-HCA2 were not less than Rs-CA2 (Fig. 4a). Unsaturated FAs constituted approximately 20% of FFAs of Rs-A2 and Rs-CA2 and twice as high a proportion in those of Rs-DCA2, Rs-AccCA2, and Rs-HCA2 (Fig. 4b). All the recombinant strains grew similarly to WT cells (Fig. 4c). Because Rs-HCA2 showed the highest FFA productivity, it was used in the subsequent experiments.

The CFA contents of *R. sphaeroides* overexpressing *plsC* alone or *plsC* with either *fabD*, *accABCD*, or *fabH* did not differ from those of WT cells (Additional file 1: Fig. S7A). The same was true for light-harvesting complexes (Additional file 1: Fig. S7B) and cell growth (Additional file 1: Fig. S7C). These results suggest that the FFAs of the recombinant strains (Fig. 4a) do not originate from the ready-made surplus membrane. The heterologous expression levels of *pla2* in Rs-CA2, Rs-DCA2, Rs-AccCA2, and Rs-HCA2 did not differ from those in Rs-A2 (Additional file 1: Table S2). Thus, it would appear that the FFA productivity of the recombinant strains is not determined by the heterologous production of PLA2 but rather by the overproduced biosynthetic enzymes, which determine the conversion rate of the PLA2-generated LPLs into PLs.

### High-cell-density culture of Rs-HCA2 grown in SisH-D medium in a two-phase system with dodecane increases FFA productivity up to 2.0 g L^−1^ day^−1^

**Fig. 5:**
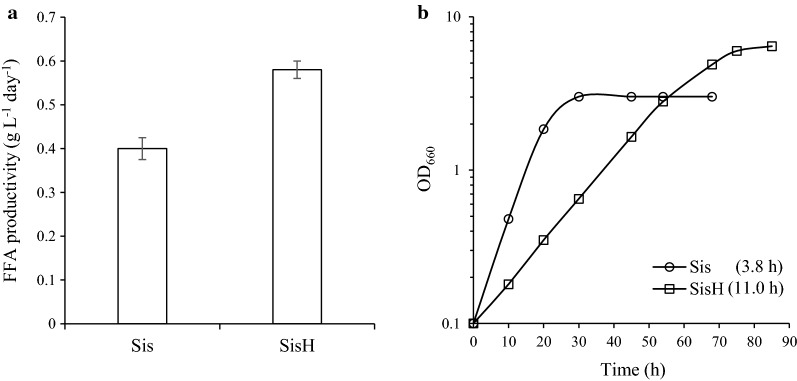
FFA productivity of Rs-HCA2 grown at high cell density. **a** Rs-HCA2 was grown photoheterotrophically in Sis minimal and SisH (Sis minimal medium supplemented with 150 mM succinate, 100 mM glucose, 150 mM glycerol, and 1% (w/v) yeast extracts) media and harvested at the exponential growth phase (OD_660_ of 2.0 in Sis minimal medium; OD_660_ of 6.0 in SisH medium). FFAs in the culture supernatants were measured, and the FFA productivity is illustrated with error bars from three independent experiments. **b** Rs-HCA2 was cultured in Sis minimal and SisH media under photoheterotrophic conditions; data shown are for one of three representative experiments

To enhance the FFA productivity of Rs-HCA2, cells were cultured at higher density in SisH medium (Sis minimal medium supplemented with 150 mM succinate, 100 mM glucose, 150 mM glycerol, and 1% (w/v) yeast extracts). The maximal turbidity of cells grown in SisH medium was almost twice that of cells grown in Sis minimal medium (Fig. 5b). Although the doubling time of Rs-HCA2 in SisH medium is threefold longer than in Sis minimal medium, the high-cell-density culture increases FFA productivity by approximately 50% (Fig. 5a).

**Fig. 6:**
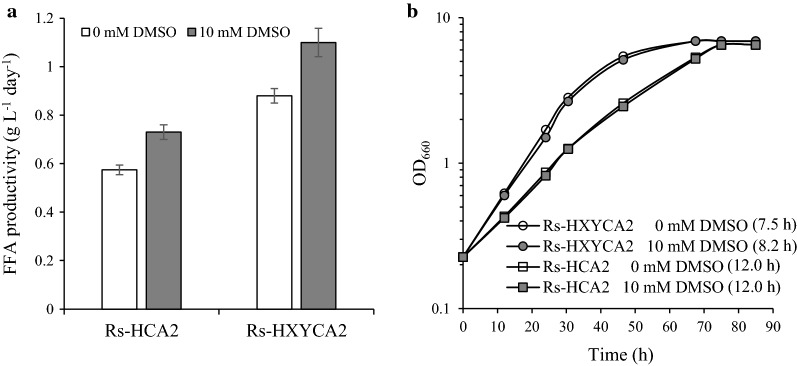
FFA productivities of Rs-HCA2 and Rs-HXYCA2 grown at high cell densities in the presence of DMSO. **a** Rs-HCA2 and Rs-HXYCA2 were grown photoheterotrophically in SisH medium with or without DMSO (10 mM) and harvested at the exponential growth phase (OD_660_ of 5.2 and 5.1 for Rs-HCA2 and Rs-HXYCA2, respectively). FFAs in culture supernatants were measured, and the FFA productivity was illustrated with error bars from three independent experiments. **b** Rs-HCA2 and Rs-HXYCA2 were cultured in SisH medium with or without DMSO (10 mM) under photoheterotrophic conditions; data shown are for one of three representative experiments

Dimethyl sulfoxide (DMSO) stimulates PL biosynthesis in yeast cells [60]. The CFA content of WT cells was reproducibly elevated by approximately 20% in several independent experiments as DMSO was increased to 10 mM (Additional file 1: Fig. S8A). At higher DMSO concentrations (≧ 25 mM), however, cell growth was affected (Additional file 1: Fig. S8C), and both CFAs (Additional file 1: Fig. S8A) and PLs (Additional file 1: Fig. S8D) decreased to lower levels than in control cells grown without DMSO. The compositions of both CFAs (Additional file 1: Fig. S8B) and PLs (Additional file 1: Fig. S8D) were not changed by DMSO. Thus, 10 mM DMSO (SisH-D medium) was used to stimulate the membrane synthesis of *R. sphaeroides*. When Rs-HCA2 was grown in SisH-D medium, the FFA productivity increased by approximately 25% (Fig. 6a) without affecting cell growth (Fig. 6b). Accordingly, Rs-HCA2 was grown photoheterotrophically in SisH-D medium to achieve higher FFA productivity in the subsequent experiments, unless otherwise specified.

FFAs produced by PLA2 in the periplasmic space may cross either the outer membrane for secretion or the inner membrane for reutilization by the cell. FFAs in the culture broth may also be utilized again after uptake by cells. Moreover, some unsaturated FAs, such as palmitoleic acid, oleic acid, linolenic acid, and arachidonic acid, affect bacterial growth by inhibiting enoyl-ACP reductase [61]. Likewise, the growth of *R. sphaeroides* was strongly affected by the presence of *cis*-vaccenate (Additional file 1: Fig. S9). Saturated FAs such as stearate and palmitate slightly slowed down the growth when present at 3 mg mL^−1^ (Additional file 1: Fig. S9A, S9B). On the other hand, the presence of *cis*-vaccenate at the same level (3 mg mL^−1^) resulted in the complete inhibition of cell growth (Additional file 1: Fig. S9C), and even a lower level (1 mg mL^−1^) of *cis*-vaccenate had a strong effect (Additional file 1: Fig. S9C). To keep these three FAs, which are the main constituents of FFAs (Fig. 4b), away from cells, a two-phase culture system with dodecane (Fig. 1) was employed. The growth of WT cells was not affected by the presence of 30% dodecane (v/v) (Additional file 1: Fig. S10). Consistently, no activity of the cytoplasmic enzyme malate dehydrogenase was detected in the culture supernatant of WT or Rs-HCA2 cells grown in the presence of 30% dodecane (Additional file 1: Fig. S11). Most enzyme activity was associated with cell lysate (Fig. S11), indicating that dodecane caused no lysis of cells. Furthermore, dodecane was shown not to be used as a sole carbon source for cell growth (Additional file 1: Fig. S10).

**Fig. 7:**
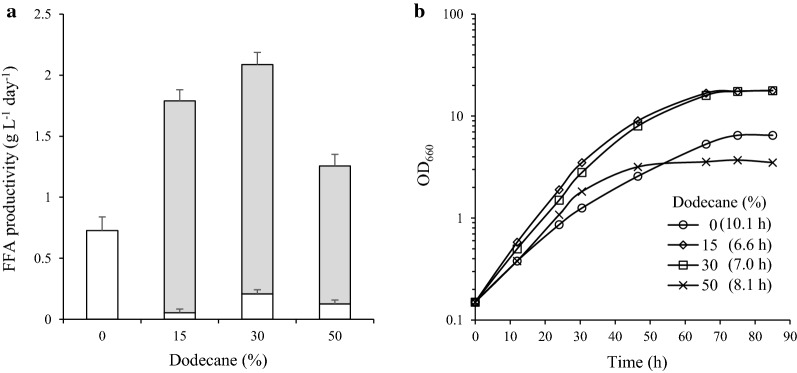
FFA productivity of Rs-HCA2 grown at high cell density in a two-phase system with dodecane. **a** Rs-HCA2 was grown photoheterotrophically in SisH-D medium. Dodecane was added to the culture at concentrations of 15%, 30%, and 50% (v/v). The culture broth and dodecane layer were harvested at the exponential growth phase (OD_660_ of 5.3 for Rs-HCA2 in the absence of dodecane; OD_660_ of 16.0, 16.0 and 3.2 for Rs-HCA2 in the presence of 15%, 30% and 50% dodecane, respectively). FFAs in the culture supernatants (open bar) and in the dodecane layer (closed bar) were measured, and the FFA productivity is illustrated with error bars from three independent experiments. **b** Rs-HCA2 was cultured in SisH-D medium in the presence of varying levels of dodecane (0, 15, 30 and 50%) under photoheterotrophic conditions; data shown are for one of three representative experiments

Rs-HCA2 grew faster in the two-phase culture system as dodecane levels increased up to 30% (Fig. 7b). The maximal cell density at 30% dodecane was threefold higher than that of cells grown without dodecane (Fig. 7b). Both the doubling time and maximal cell density were affected at higher (50%) dodecane levels (Fig. 7b). The FFA productivity of Rs-HCA2 was the highest, approximately 2.0 g L^−1^ day^−1^, at 30% dodecane (Fig. 7a). More than 90% of FFAs were localized to the dodecane layer (Fig. 7a), and there was no difference in composition between the FFAs in the culture broth and those in the dodecane layer (Additional file 1: Fig. S12). Thus, FFA productivity was elevated by keeping the FFAs in the dodecane layer. Accordingly, Rs-HCA2 was cultured in a two-phase system with 30% dodecane to increase FFA productivity in the subsequent experiments, unless otherwise specified.

The FFAs produced by Rs-HCA2 (Fig. 4b, Additional file 1: Fig. S12) consist mainly of *cis*-vaccenate, stearate, and palmitate in an approximately equimolar ratio. As in the FFAs produced by Rs-A2 (Fig. 4), the lower-than-expected proportion of *cis*-vaccenate among the FFAs indicates its importance in the membrane for the viability and growth of *R. sphaeroides*. The proportion of *cis*-vaccenate in the CFAs of Rs-HCA2 is approximately 60% (Additional file 1: Fig. S13A), which is less than that in WT cells by 10% (Additional file 1: Fig. S13A). The sum of stearate and palmitate comprises the remaining 40% of CFAs (Fig. S13A). The reason for the change in the CFA composition of Rs-HCA2 remains to be determined, but it would appear that the lower unsaturated FA in the Rs-HCA2 membranes might reflect the response to stress from the hydrolysis of the membrane by PLA2. The PL composition in Rs-HCA2, however, was not different from that of WT cells (Additional file 1: Fig. S13B).

### Overproduction of both PlsX and PlsY in Rs-HCA2, generating Rs-HXYCA2, leads to accelerated cell growth

The expression levels of *fabH* and *plsC* in Rs-HCA2 were examined by RT-qPCR and shown as the mean ratio to the expression of the corresponding gene in WT cells (Table 2). As expected, both *fabH* and *plsC* were overexpressed in Rs-HCA2 compared with WT cells. Interestingly, *plsX* and *plsY* of Rs-HCA2, which encode phosphate acyltransferase and glycerol-3-phosphate acyltransferase, respectively, catalyzing the two consecutive metabolic steps immediately upstream of PlsC (Fig. 1), were expressed to a lesser extent than in WT cells (Table 2). The transcriptional network between *plsX*, *plsY*, and *plsC* in the presence of extra copies of *pla2* remains to be defined, but the overexpression of *plsC* in Rs-HCA2 may result in a higher demand for acyl-ACP, which is also a substrate of PlsX (Fig. 1). As a result, the steady-state level of acyl-ACP may be lowered, which in turn may affect the expression of *plsX* and *plsY* (Fig. 1).

**Table 2:** Expression of *fabH*, *plsX*, *plsY*, *plsC*, and *pla2* in Rs-HCA2 and Rs-HXYCA2

Genes^b^	Strains^a^		
	WT	Rs-HCA2	Rs-HXYCA2
*fabH*	1.0 ± 0.7	64.7 ± 0.2^d^ (0.007)^e^	52.4 ± 0.1^d^ (0.032)^e^
*plsX*	1.0 ± 0.1	0.6 ± 0.1^d^ (0.04)^e^	15.1 ± 0.7^d^ (0.001)^e^
*plsY*	1.0 ± 0.1	0.2 ± 0.1^d^ (0.012)^e^	89.0 ± 0.7^d^ (0.001)^e^
*plsC*	1.0 ± 0.1	6.4 ± 0.4^d^ (0.004)^e^	6.2 ± 0.2^d^ (0.004)^e^
*pla2*	NA^c^	1.0 ± 0.4	1.1 ± 0.1^f^ (0.3)^e^

^a^Cells were grown photoheterotrophically in SisH medium and harvested at the exponential phase (OD_660_ of 9.5, 5.3 and 5.4 for WT, Rs-HCA2 and Rs-HXYCA2, respectively)

^b^Expression levels were examined by RT-qPCR

^c^Not applicable

^d^Mean ratio (± SD) to the expression of the corresponding gene in WT cells, which was set to 1, from three independent experiments with 16S rRNA as a reference gene

^e^Significance level regarded as significant when the value is less than 0.05

^f^Mean ratio (± SD) to the expression of *pla2* in Rs-HCA2, which was set to 1, from three independent experiments with 16S rRNA as a reference gene

To elevate the low expression of *plsX* and *plsY*, both genes were overexpressed in Rs-HCA2, generating the strain Rs-HXYCA2 (Table 2). RT-qPCR analysis revealed elevated expression of both *plsX* and *plsY* in Rs-HXYCA2 compared with the corresponding expression levels in Rs-HCA2 (Table 2). The expression of *pla2* remained unchanged in the same comparison (Table 2). Interestingly, Rs-HXYCA2 grew faster than Rs-HCA2, irrespective of the presence of DMSO (Fig. 6b). The CFA composition of Rs-HXYCA2 was not different from that of Rs-HCA2 (Additional file 1: Fig. S13A). The same was true for the PLs of these two strains (Additional file 1: Fig. S13B). Thus, it would appear that the overexpression of *plsX* and *plsY* in Rs-HCA2 led to the efficient synthesis of phosphatidate as a precursor of PL and subsequently to accelerated cell growth. The FFA productivity of Rs-HXYCA2 was approximately 40% higher than that of Rs-HCA2 (Fig. 6a), and the addition of 10 mM DMSO to the culture broth stimulated the FFA productivity of Rs-HXYCA2 to an extent similar to that observed in Rs-HCA2 (Fig. 6a).

### High-cell-density culture of Rs-HXYCA2 in SisH-D medium in a two-phase system with dodecane further elevates FFA productivity up to 2.8 g L^−1^ day^−1^ with an FFA titer of approximately 8.5 g L^−1^

**Fig. 8:**
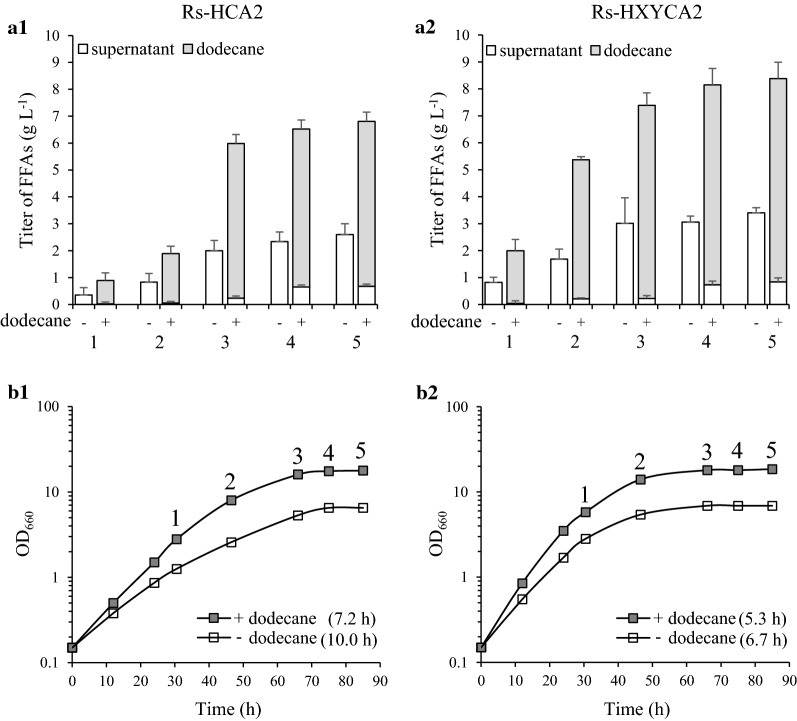
FFA productivities of Rs-HCA2 and Rs-HXYCA2 grown at high cell densities in a two-phase system with dodecane. **a1** Rs-HCA2 and **a2** Rs-HXYCA2 were grown photoheterotrophically in SisH-D medium with or without 30% dodecane (v/v). The culture broth and dodecane layer were harvested during growth, and the FFAs in the culture supernatant (open bar) and the dodecane layer (closed bar) were measured. The FFA productivity is illustrated with error bars from three independent experiments. The numbers 1–5 correspond to the time points of harvesting the culture broth and dodecane layer from **b1** and **b2**. **b1** Rs-HCA2 and **b2** Rs-HXYCA2 were cultured in SisH-D medium with or without 30% dodecane (v/v) under photoheterotrophic conditions; data shown are for one of three representative experiments

We then examined the FFA productivity of Rs-HXYCA2 grown in SisH-D medium in a two-phase culture system with dodecane (Fig. 8a2, b2). Rs-HCA2 was grown in the same conditions and examined as a control (Fig. 8a1, b1). The FFA productivity of Rs-HCA2 in a two-phase culture system with dodecane was 2.0 g L^−1^ day^−1^ with a maximal FFA titer of 6.8 g (Fig. 8a1 and Table 3). When Rs-HCA2 was grown without dodecane, the FFA productivity and the maximal FFA titer were approximately 40% of the corresponding values in the cells grown with dodecane (Fig. 8a1 and Table 3).

**Table 3:** Carbon-to-lipid conversion yield, FFA titer, and FFA productivity of Rs-HCA2 and Rs-HXYCA2

	Rs-HCA2		Rs-HXYCA2	
	Dodecane			
	–	+	–	+
Carbon-to-lipid conversion yield (g g^−1^)^a^	0.14 ± 0.02^d^	0.17 ± 0.01	0.18 ± 0.01	0.20 ± 0.01
Titer (g L^−1^)^b^	2.6 ± 0.2	6.8 ± 0.5	3.4 ± 0.5	8.5 ± 0.7
Productivity (g L^−1^ day^−1^)^c^	0.7 ± 0.2	2.0 ± 0.1	1.0 ± 0.2	2.8 ± 0.2

^a^Ratio of total lipids to the carbon sources (glucose, glycerol, and succinate) consumed by the recombinant strains (Additional file 1: Fig. S16), calculated as described previously [62]

^b^Maximum FFA level per liter of culture medium

^c^Productivity at the exponential growth phase (OD_660_ of 6.5 and 17.5 for Rs-HCA2 in the absence and the presence of dodecane, respectively; OD_660_ of 6.9 and 14.0 for Rs-HXYCA2 in the absence and the presence of dodecane, respectively)

^d^Mean ± SD

Rs-HXYCA2 grown in SisH-D medium in a two-phase culture system with dodecane showed higher FFA productivity of 2.8 g L^−1^ day^−1^ with a maximal FFA titer of approximately 8.5 g L^−1^ (Fig. 8a2 and Table 3). The FFA productivity and maximal FFA titer without dodecane were also approximately 40% of the corresponding values in the cells grown with dodecane (Fig. 8a2 and Table 3). Rs-HXYCA2 grew faster than Rs-HCA2 irrespective of the presence of dodecane (Fig. 8b1, b2). As observed with Rs-HCA2 (Fig. 7 and Fig. 8a1), more than 90% of FFAs were localized to the dodecane layer (Fig. 8a2). There was no difference in compositions between the FFAs in the culture broth and those in the dodecane (Additional file 1: Fig. S12). Furthermore, the FFA composition did not vary during growth of Rs-HCA2 and Rs-HXYCA2 (Additional file 1: Fig. S14). Interestingly, the expression levels of the genes carried on plasmids were elevated when Rs-HXYCA2 was grown with dodecane, compared with those in cells grown without dodecane (Additional file 1: Fig. S15, A2, B2, C2 and D2). The same was true for Rs-HCA2 (Additional file 1: Fig. S15, A1, B1, C1 and D1). Thus, keeping the FFAs in dodecane results in enhanced cell growth and higher FFA productivity, which is consistent with the elevated expression of genes encoding enzymes for the formation of CFAs and phosphatidate.

The residual carbons in the culture broth were determined (Additional file 1: Fig. S16), and the conversion yields from carbon to lipid by Rs-HCA2 and Rs-HXYCA2 were calculated as described previously [62]. Rs-HCA2 showed conversion yields in the range from 0.14 without dodecane to 0.17 with dodecane (Table 3). The conversion yields of Rs-HXYCA2 were in the range from 0.18 without dodecane to 0.20 with dodecane (Table 3). Thus, although the overexpression of *plsX* and *plsY* in Rs-HCA2 together with the sequestration of FFAs in the dodecane layer had a positive effect on the carbon-to-lipid conversion yield, the increase of FFA productivity in the two-phase system is due to an increase in overall carbon substrate consumption rather than due to an increased efficiency of carbon substrate conversion to FFA (Table 3, Fig. 8 and Additional file 1: Fig. S16).

In summary, the photosynthetic bacteria *R. sphaeroides* can be used as an ideal oleaginous host source for the production of long-chain FFAs based on its intrinsic high lipid contents. Because *R. sphaeroides* is capable of using various carbon and nitrogen sources as substrates to grow in a wide variety of conditions, additional genetic modification can also be applied to enhance the potential of this bacterium for the production of FFAs.

## Additional file

**Additional file 1.** Additional figures and tables.
