# Supplementary material for: Production of long-chain free fatty acids from metabolically engineered Rhodobacter sphaeroides heterologously producing periplasmic phospholipase A2 in dodecane-overlaid two-phase culture
Source: Microb Cell Fact. 2019 Jan 31;18:20. doi: 10.1186/s12934-019-1070-8 (PMC6357386; doi:10.1186/s12934-019-1070-8)
Supplement: Supplementary file 1 — Additional file 1. Additional figures and tables. [file 12934_2019_1070_MOESM1_ESM.pdf]

Table S1. PCR primers used in this study

Primer	Sequence (5'-3')	Notes
AccA-F	GC**T CTA GA**T TCC TCC GGC GCT CGT GTG G	*Xba*I is in boldface
AccA-R	AA**C TGC AG**T CAC GCC GCG AGG CCC TTC GAC	*Pst*I is in boldface
AccBC-F	C**AA GCT T**GT TGT CCG GCG TCT TGT AGC	*Hin*dIII is in boldface
AccBC-R	GC**T CTA GA**C GGC GAA ATC CTC ATC CAC	*Xba*I is in boldface
AccD-F	GC**T CTA GA**G TCT CCT CCG AAG CAG GCG C	*Xba*I is in boldface
AccD-R	AA**C TGC AG**T CAC TCG GCG GCC TTG TCG TCG	*Pst*I is in boldface
CycA-F	C**CC ATG G**AT GAA GTT CCA AGT C	*Nco*I is in boldface
CycA-R	C**TC TAG A**CG CGA GCG CCG GCA GCG CCG CGA AT	*Xba*I is in boldface
FabD-F	GC**T CTA GAA** GGA TCA TCA TAT CCG CAC AGT TC	*Xba*I is in boldface
FabD-R	AA**C TGC AG**T CAG GCC GTG GCG GCA GCC GC	*Pst*I is in boldface
FabH-F	GG**G GTA CC**C TAT GCC CGC AAC GGT CTC AGC CTC	*Kpn*I is in boldface
FabH-R	GC**T CTA GA**T GAA GCA CGG ATT CCA CCA G	*Xba*I is in boldface
PhoA-F	C**CT GCA G**GC TCA GGG CGA TAT TAC TGC A	*Pst*I is in boldface
PhoA-R	C**AA GCT T**TT ATT TCA GCC CCA GAG CGG	*Hin*dIII is in boldface
PLA1-F	GC**T CTA GA**A TGG CGA CCA TTC CCT CCC AC	*Xba*I is in boldface
PLA1-R	C**AA GCT T**TT ACA AGA GTT GTG ATG GAT G	*Hin*dIII is in boldface
PLA2-F	C**CC ATG G**CT TAA CGT CGG TGT TCA GCT CAT	*Nco*I is in boldface
PLA2-F´	C**TC TAG A**CT TAA CGT CGG TGT TCA GCT CAT	*Xba*I is in boldface
PLA2-R	C**AA GCT T**TT AGG GTT TCT TGA GGA CTT TGC CG	*Hin*dIII is in boldface
PLA2-t-F	CAT G**CC ATG G**CT TAA CGT CGG TGT TCA GCT	*Nco*I is in boldface
PLA2-t-R	G**CT GCA G**GG GTT TCT TGA GGA CTT TGC CG	*Pst*I is in boldface

PlsC-F	C**TC TAG A**GC CTC GTT GCC GTG ATG CTC CTG	*Xba*I is in boldface
PlsC-R	G**GG TAC C**GG GCT TCT GCG CTA CGT CTT CGG	*Kpn*I is in boldface
PlsX-F	CCC **AAG CTT** CGT CGC TTC CAA TCC GGC CGA G	*Hin*dIII is in boldface
PlsX-R	GC**T CTA GA**A GTT CGG GAC GAC ACG GTC G	*Xba*I is in boldface
PlsY-F	C**GG TAC C**GT CCT CGC CCG GGC CTG TCA TTC AC	*Kpn*I is in boldface
PlsY-R	G**GA ATT C**CG CGG ACG AGC CCT CGA AGG GTT	*Eco*RI is in boldface
TolC-UF	C**CT GCA G**AC GTG GTG GTG GCG CCG AAC CG	*Pst*I is in boldface
TolC-UR	C**GG ATC C**CA TTG CCC TCT CCA CTG TCC CGT	*Bam*HI is in boldface
TolC-DF	C**AA GCT T**TG AGA CTT TCC TTC CTG CGG GGC A	*Hin*dIII is in boldface
TolC-DR	C**GA ATT C**GC CGT CTC AGA CGC GCG CGA AGG	*Eco*RI is in boldface
Rt-FabH-F	CTT GCA GAA ACA GCG CAT AC	
Rt-FabH-R	AAG GTC GCC TTC CTT GAT CT	
Rt-PlsC-F	CAC CAG AGC TTC CTC GAT TC	
Rt-PlsC-R	GAC ATC GGC CAT CAT CTT CT	
Rt-PlsX-F	GTG GTC ACG ATG AAC GAC AA	
Rt-PlsX-R	GAT CAT CGA GAC TGC CAT CA	
Rt-PlsY-F	GAG TGC TGG GCT ATC TGC TC	
Rt-PlsY-R	CCC AGA AAC GAG GTG AAG G	
Rt-PLA2-F	CAG TGG ATG TCC TGG TGA GA	
Rt-PLA2-R	GCG TCG CAT TTG TTA CCT TT	
Rt-16S-F	CAG CTC GTG TCG TGA GAT GT	
Rt-16S-R	TAG CAC GTG TGT AGC CCA AC	

Table S2. Expression of *pla2*[a] in the recombinant *R. sphaeroides* strains[b].

Rs-A2	Rs-CA2	Rs-DCA2	Rs-AccCA2	Rs-HCA2
1.0 ± 0.2	1.0 ± 0.1[c]	1.0 ± 0.1	1.1 ± 0.1	1.1 ± 0.2

[a]Expression of *pla2* was examined by RT-qPCR.
[b]Cells were grown photoheterotrophically in Sis minimal medium and harvested at the exponential growth phase (OD_{660} of 2.3, 2.3, 2.4, 2.3 and 2.2 for Rs-A2, Rs-CA2, Rs-DCA2, Rs-AccCA2 and Rs-HCA2, respectively).
[c]Mean ratio (± SD) with respect to Rs-A2 from three independent experiments with 16S rRNA as a reference gene.

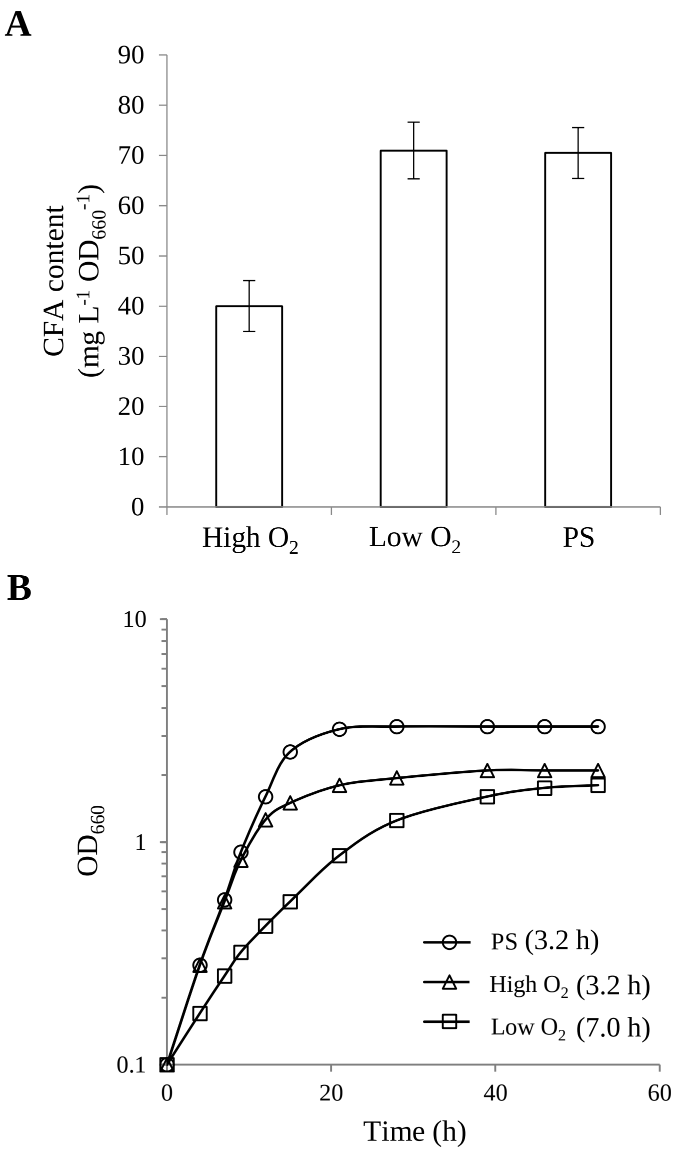

Figure S1. Total cellular FA (CFA) content and growth profile of *R. sphaeroides* KD131 under aerobic, semiaerobic, and photoheterotrophic conditions. (A) *R. sphaeroides* KD131 was grown in Sis minimal medium under aerobic (High O_2), semiaerobic (Low O_2), and photoheterotrophic (PS) conditions, and cells were harvested at the exponential growth phase (optical density at 660 nm (OD_{660}) of 0.8, 0.9 and 1.6, respectively). The total CFA content is illustrated with error bars from three independent experiments. (B) Growth profiles of *R. sphaeroides* under the three different conditions are shown. Doubling time is shown in parentheses. The results shown are for one of three representative growth experiments.

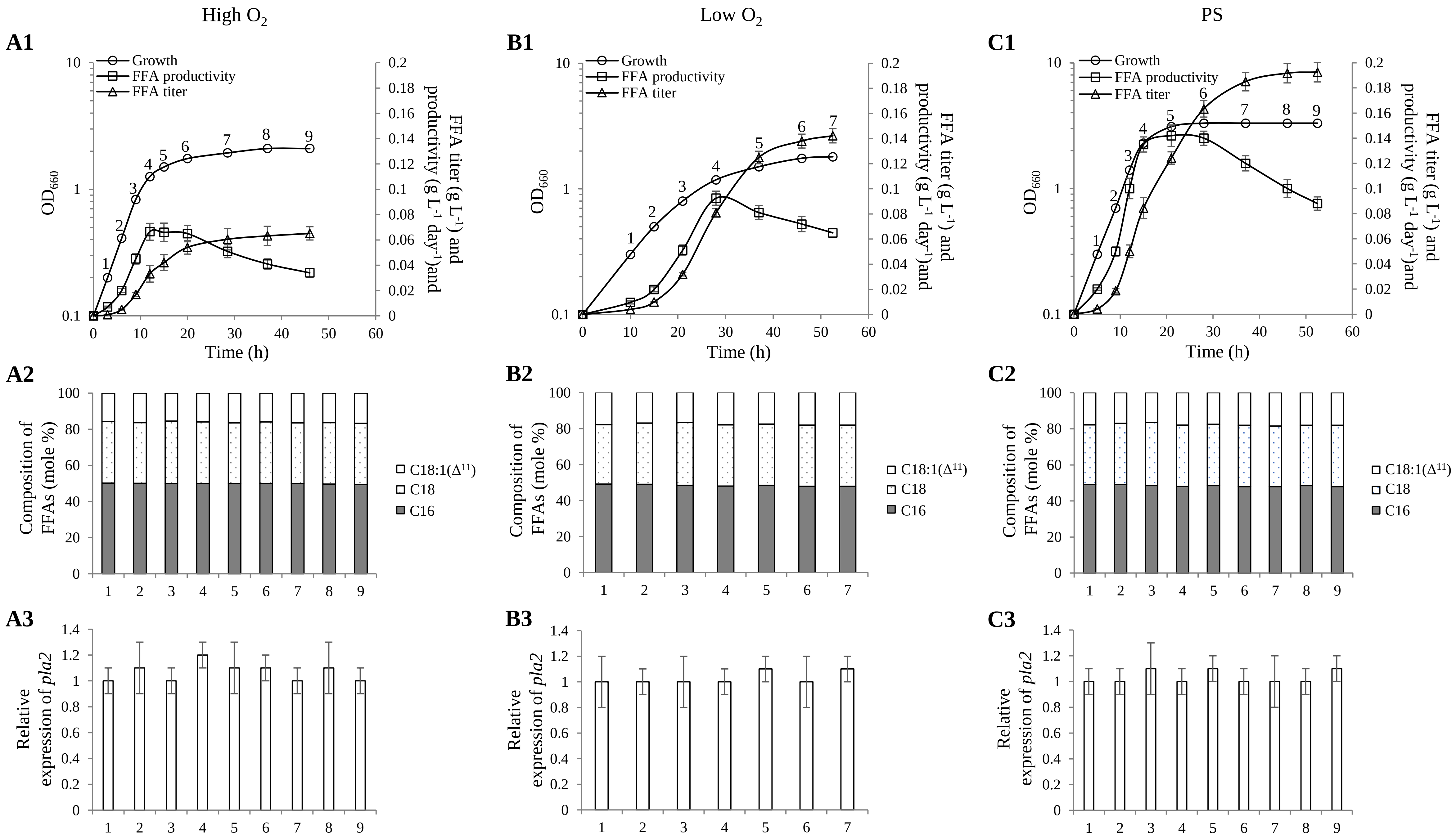
High O2
Low O2
PS
A1
B1
C1
Growth
FFA productivity
FFA titer
OD660
FFA titer (g L-1) and productivity (g L-1 day-1)and
Time (h)
A2
B2
C2
Composition of FFAs (mole %)
C18:1(Δ11)
C18
C16
A3
B3
C3
Relative expression of pla2

Figure S2. FFA productivity of Rs-A2 grown under aerobic, semiaerobic, and photoheterotrophic conditions. Rs-A2 was grown in Sis minimal medium under aerobic (High O_2) (A1), semiaerobic (Low O_2) (B1), and photoheterotrophic (PS) (C1) conditions, and culture aliquots were harvested during growth. The numbers 1 to 9 (for A and C) or 1 to 7 (for B) correspond to the time points of harvesting the culture broth. FFAs in the culture supernatant were determined, and the FFA titers and productivities are illustrated with error bars from three independent experiments (A2, B2 and C2). Expression of *pla2* was examined by RT-qPCR (A3, B3 and C3), and the mean ratios (± SD) of *pla2* expressions at time points 2 to 9 (A3 and C3) or 2 to 7 (B3) to the expression level of *pla2* at time point 1, which was set to 1, were determined from three independent experiments with 16S rRNA as a reference gene.

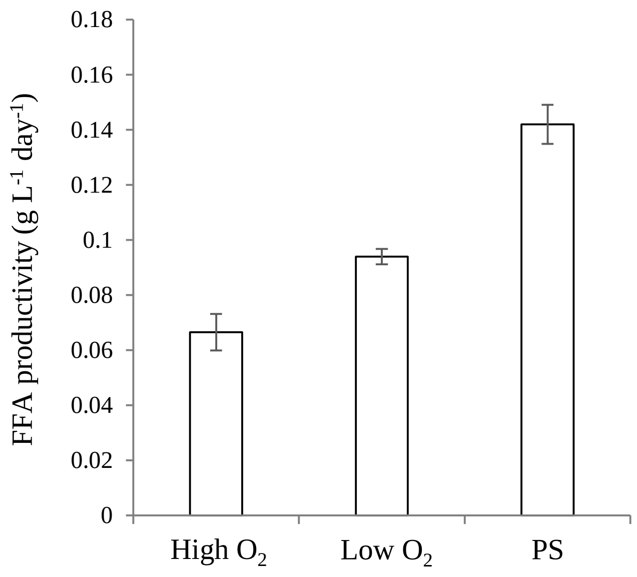

Figure S3. Summary of FFA productivity of Rs-A2 grown under aerobic, semiaerobic, and photoheterotrophic conditions. Rs-A2 was grown in Sis minimal medium under aerobic (High O_2), semiaerobic (Low O_2), and photoheterotrophic (PS) conditions as shown in Fig. S2, and culture aliquots were harvested at the end of exponential growth phase (OD_{660} of 1.5 for High O_2, OD_{660} of 1.2 for Low O_2 and OD_{660} of 2.3 for PS). FFAs in the culture supernatant were determined, and the representative FFA productivities of Rs-A2 are illustrated with error bars from three independent experiments.

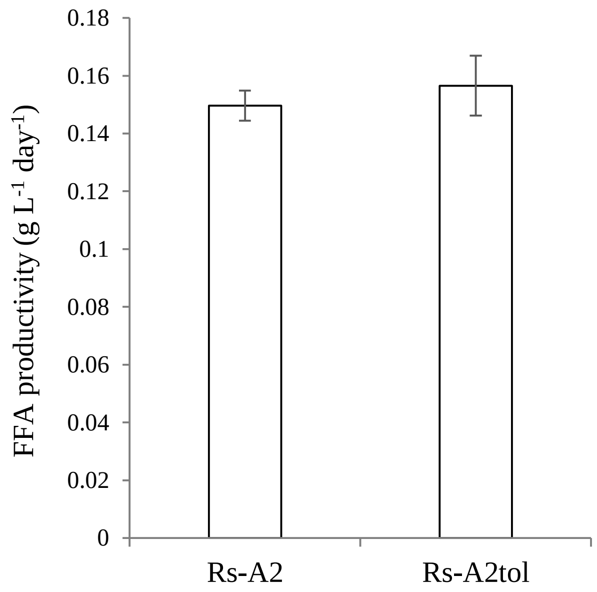

Figure S4. FFA productivity of Rs-A2 and Rs-A2tol grown under photoheterotrophic conditions. Rs-A2 and Rs-A2tol were grown photoheterotrophically in Sis minimal medium, and cells were harvested at the exponential growth phase (OD_{660} of 2.3 and 2.2 for Rs-A2 and Rs-A2tol, respectively). FFAs in the culture supernatant were determined and are illustrated with error bars from three independent experiments.

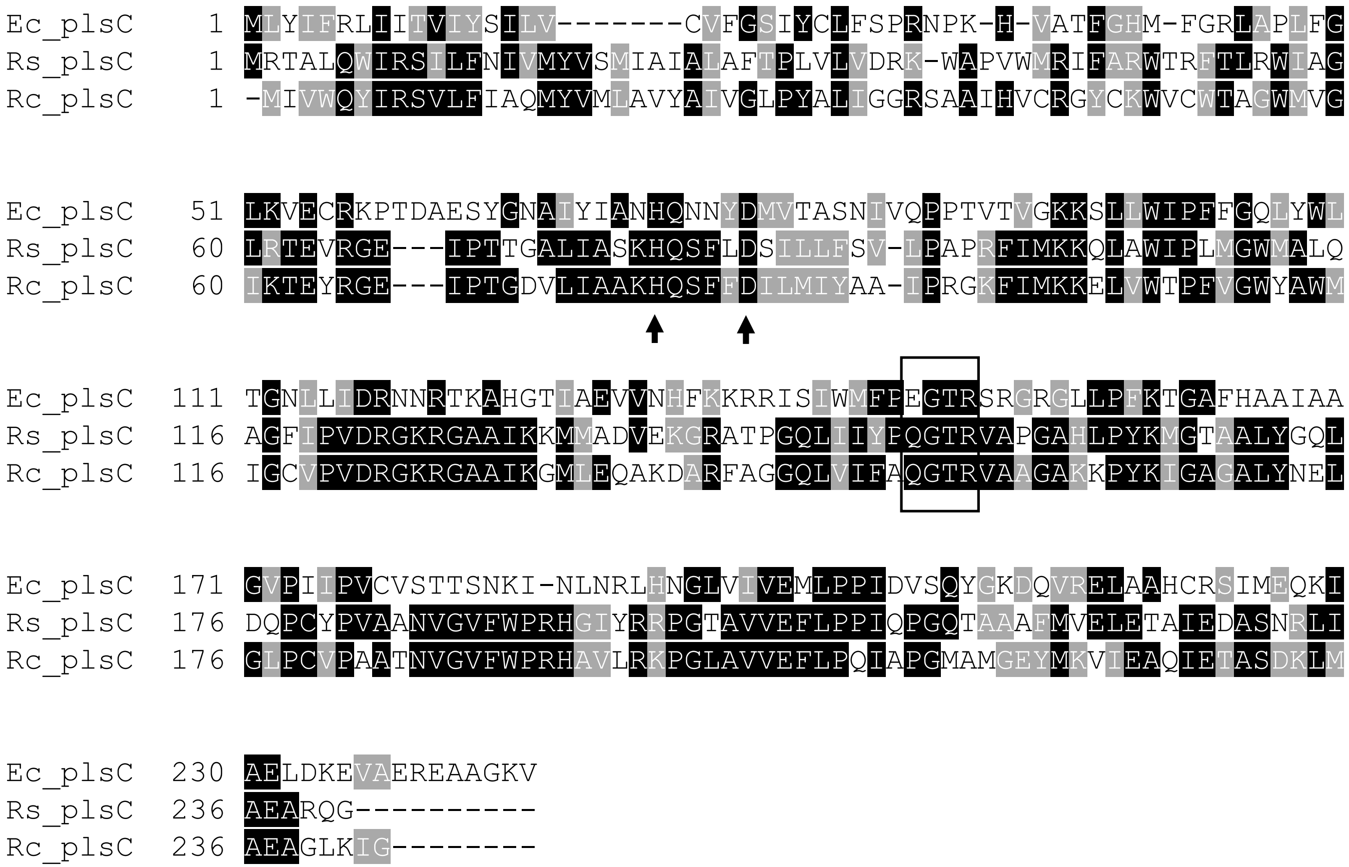

Figure S5. Sequence alignment of PlsCs. The amino acid sequences of PlsCs from *E. coli*, *R. sphaeroides* and *R. capsulatus* were aligned using the software BOXSHADE (version 3.21). Identical residues are shaded in black, and similar residues are shaded in gray. The residues corresponding to the conserved catalytic motif (HX_4D) and the substrate binding motif (EGTR) of *E. coli* PlsC are indicated by two arrows and a box, respectively.

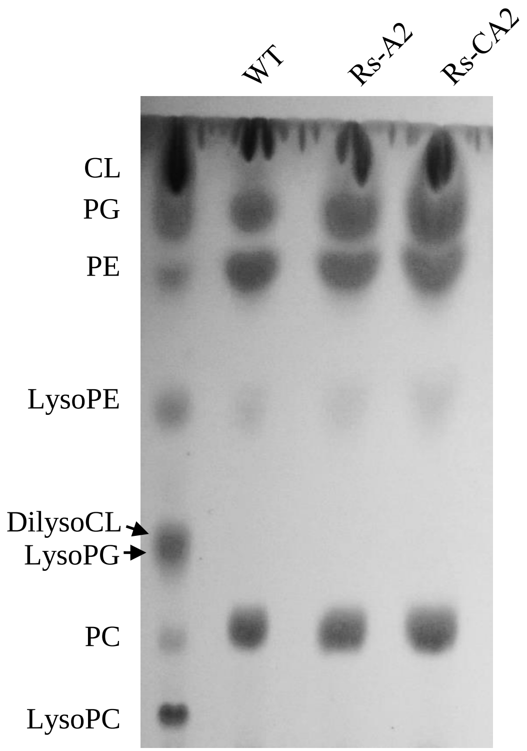

Figure S6. Cellular PL compositions of Rs-A2 and Rs-CA2 grown under photoheterotrophic conditions. Rs-A2 and Rs-CA2 were grown photoheterotrophically in Sis minimal medium and harvested at the exponential growth phase (OD_{660} of 2.5, 2.3 and 2.2 for WT, Rs-A2 and Rs-CA2, respectively). WT cells were included as a control. Membrane lipids were extracted from WT, Rs-A2, and Rs-CA2 cells, and aliquots (30 µg each) were subjected to TLC analysis. To determine the migration profiles of LPLs, membrane lipids of WT cells (400 µg) were digested by 5 µg of honey bee venom PLA2 (Sigma-Aldrich) as described in section 2.7 (leftmost lane). LPLs from Sigma-Aldrich were used to localize the positions of lysoPE, dilysoCL, lysoPG, and lysoPC. CL, cardiolipin; PG, phosphatidylglycerol; PE, phosphatidylethanolamine; PC, phosphatidylcholine. Sulfoquinovosyl diacylglycerols (SQDGs) are not a substrate for PLA2. Furthermore, SQDGs were not detected under this TLC condition when developed with a mixture of chloroform-methanol-acetic acid-acetone-water (35:25:4:14:2, vol/vol) [49]. The experiments were independently repeated three times; the data shown are for one of three representative experiments.

A

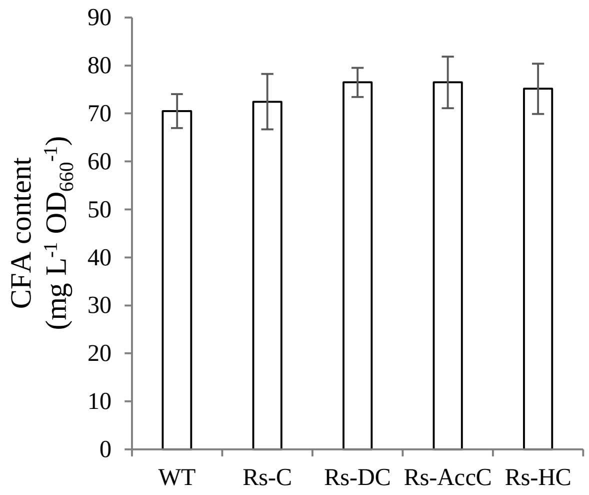

B

Light-harvesting complexes
(nmole/mg protein)

Strains	B800-850[a]	B875[b]
WT	7.0 ± 0.7	6.0 ± 0.3
Rs-C	7.4 ± 0.4	6.1 ± 0.4
Rs-DC	6.8 ± 1.0	5.7 ± 0.6
Rs-AccC	6.8 ± 1.1	5.8 ± 0.4
Rs-HC	7.1 ± 0.5	6.1 ± 0.2

[a] $A_{849-900}$ with ε of 96 mM^{-1} cm^{-1}.
[b] $A_{878-820}$ with ε of 73 mM^{-1} cm^{-1}.

C

Strain	WT	Rs-C	Rs-DC	Rs-AccC	Rs-HC
Doubling time (h)	3.0	3.0	3.1	3.1	3.1

All these cells grew to OD_{660} values of approximately 3.2.

Figure S7. CFA content, light-harvesting complex level, and growth of recombinant *R. sphaeroides* strains Rs-C, Rs-DC, Rs-AccC, and Rs-HC under photoheterotrophic conditions. (A) Cells were grown photoheterotrophically in Sis minimal medium and harvested at the exponential growth phase (OD_{660} of 2.5, 2.5, 2.5, 2.6 and 2.6 for WT, Rs-C, Rs-DC, Rs-AccC and Rs-HC, respectively). CFA content was determined and is shown with error bars from three independent experiments. WT cells were included as a control. (B) The levels of light-harvesting complexes of the recombinant *R. sphaeroides* strains grown and harvested as described in (A) were determined and are shown as the mean ± SD from three independent experiments. (C) Doubling times of recombinant *R. sphaeroides* strains were determined and are shown with WT cells as a control. The growth data presented here were reproduced within SDs of 10-15%. All the experiments were independently repeated three times.

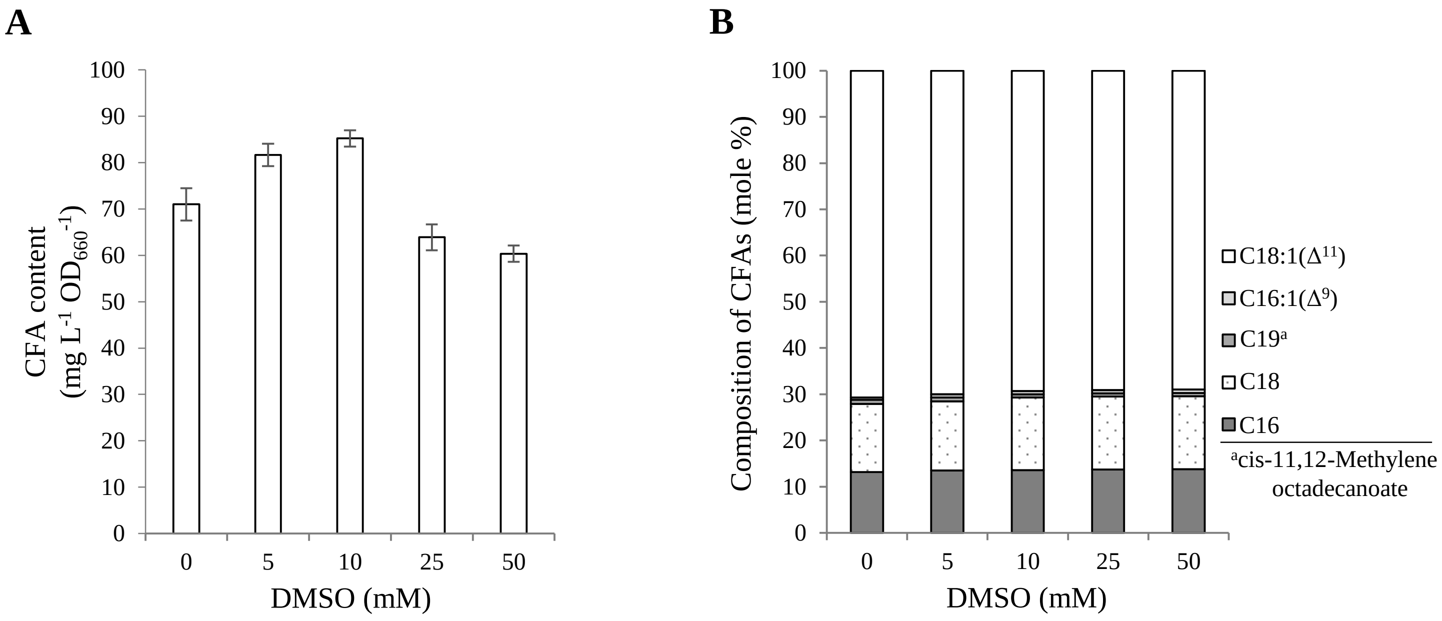

C

DMSO (mM)	0	5	10	25	50
Doubling time (h)	3.0	3.0	3.2	4.0	4.3
Maximal OD_{660}	3.2	3.2	3.2	2.8	2.6

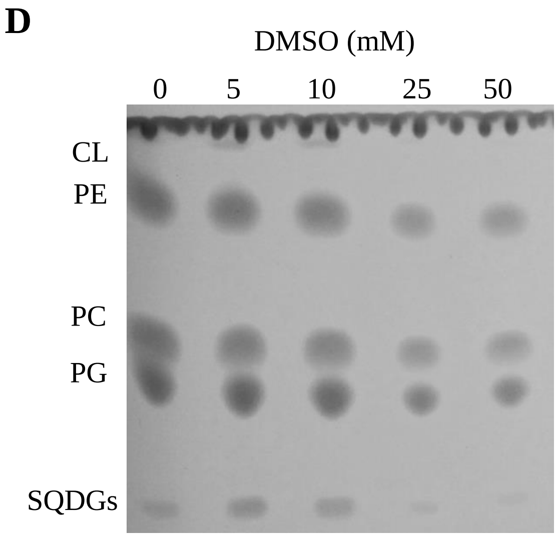

Figure S8. CFA content and CFA and cellular PL compositions of *R. sphaeroides* WT cells grown in the presence of DMSO under photoheterotrophic conditions. (A) WT cells were grown photoheterotrophically in Sis minimal medium supplemented with varying levels of DMSO and harvested at the exponential growth phase (OD_{660} of 2.6). CFA content was determined and is shown with error bars from three independent experiments. (B) CFA compositions of WT cells grown and harvested as described in (A) were determined. (C) Doubling time and maximal growth of cells cultured at varying levels of DMSO. The growth data presented here were reproduced within SDs of 10-15%. (D) Membrane lipids of WT cells grown and harvested as described in (A) were prepared, and aliquots (30 µg each) were analyzed by TLC. All the results shown here were similarly reproduced in three independent experiments, and the data shown are for one of three representative experiments. CL: Cardiolipin, PE: phosphatidylethanolamine, PC: phosphatidylcholine, PG: phosphatidylglycerol, SQDGs: sulfoquinovosyl diacylglycerols.

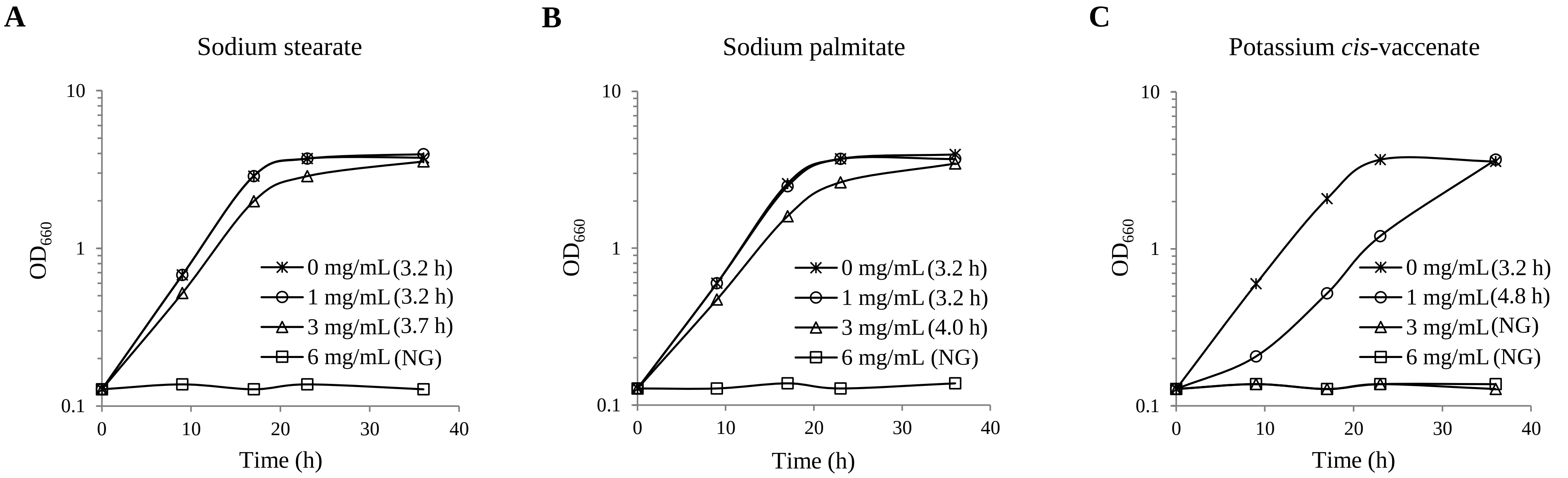

Figure S9. Effect of exogenous FAs on the growth of *R. sphaeroides* WT cells under photoheterotrophic condition. WT cells were grown photoheterotrophically in Sis minimal medium supplemented with varying levels of FFAs: sodium stearate (A), sodium palmitate (B) and potassium *cis*-vaccenate (C). The experiments were independently repeated three times; data shown are for one of three representative experiments.

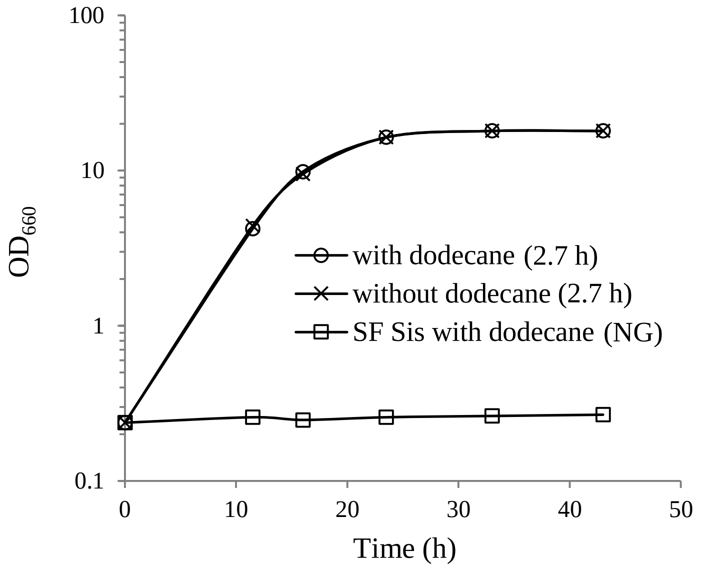

Figure S10. Effect of dodecane on the growth of *R. sphaeroides* WT cells under photoheterotrophic condition. WT cells were grown photoheterotrophically in SisH medium with (open circle) or without (cross) 30% dodecane. No cell growth was observed in succinate-free Sis minimal medium (SF Sis) supplemented with 30% dodecane (open box), which indicates that dodecane is not used as a sole carbon source. The results shown here were similarly reproduced in three independent experiments, and the data shown are for one of three representative experiments. NG stands for no growth.

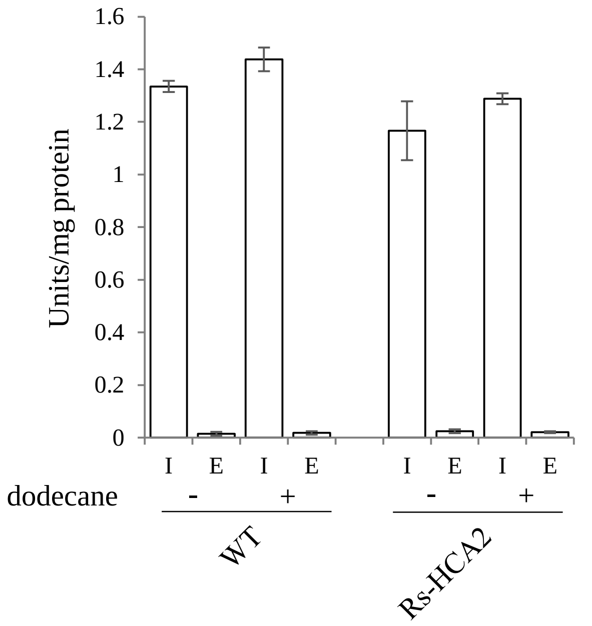

Figure S11. Malate dehydrogenase activities of *R. sphaeroides* WT cells and Rs-HCA2. Cells were grown photoheterotrophically in SisH-D medium with (+) or without (-) dodecane (30%, v/v). Aliquots (10 mL) of cell culture were harvested at the exponential growth phase (OD_{660} of 9.5 for WT in the absence and the presence of dodecane, respectively; OD_{660} of 5.4 and 16.0 for Rs-HCA2 in the absence and the presence of dodecane, respectively), and cell-free lysate was prepared as described in section 2.11. Culture supernatant was concentrated approximately tenfold using Amicon® Ultra-4 centrifugal Filters-3K (Merck, Ireland). Malate dehydrogenase activities in both intracellular (I: cell lysate) and extracellular (E: culture supernatant) fractions from 10 mL of culture were determined and shown as the mean ± SD from three independent experiments. WT cells were included as a control. Extracellular activities are less than 1% of the total activities, which are the sum of intracellular and extracellular activities. One unit of malate dehydrogenase activity is defined as the amount of the enzyme that produces 1 µmole of product per min under the conditions specified in section 2.11.

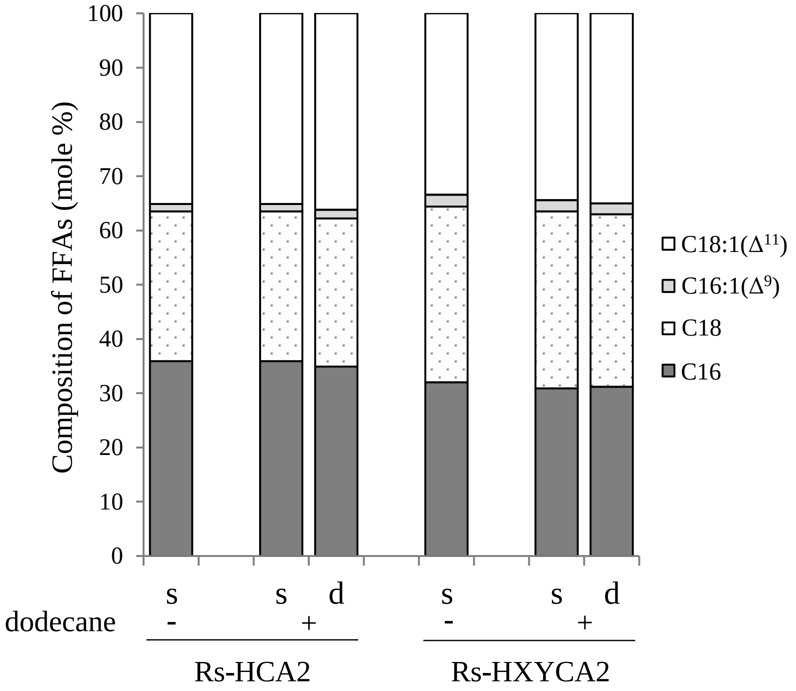

Figure S12. FFA compositions of Rs-HCA2 and Rs-HXYCA2. Cells were grown photoheterotrophically in SisH-D medium with (+) or without (-) dodecane (30%, v/v). Cells were harvested at the exponential growth phase (OD_{660} of 5.3 and 5.4 for Rs-HCA2 and Rs-HXYCA2 in the absence of dodecane, respectively; OD_{660} of 16.0 and 14.0 for Rs-HCA2 and Rs-HXYCA2 in the presence of dodecane, respectively), and the FFAs in the culture supernatant (s) and dodecane layer (d) were determined from three independent experiments. The results were reproduced within SDs of 10-15%, and the data shown are for one of three representative experiments.

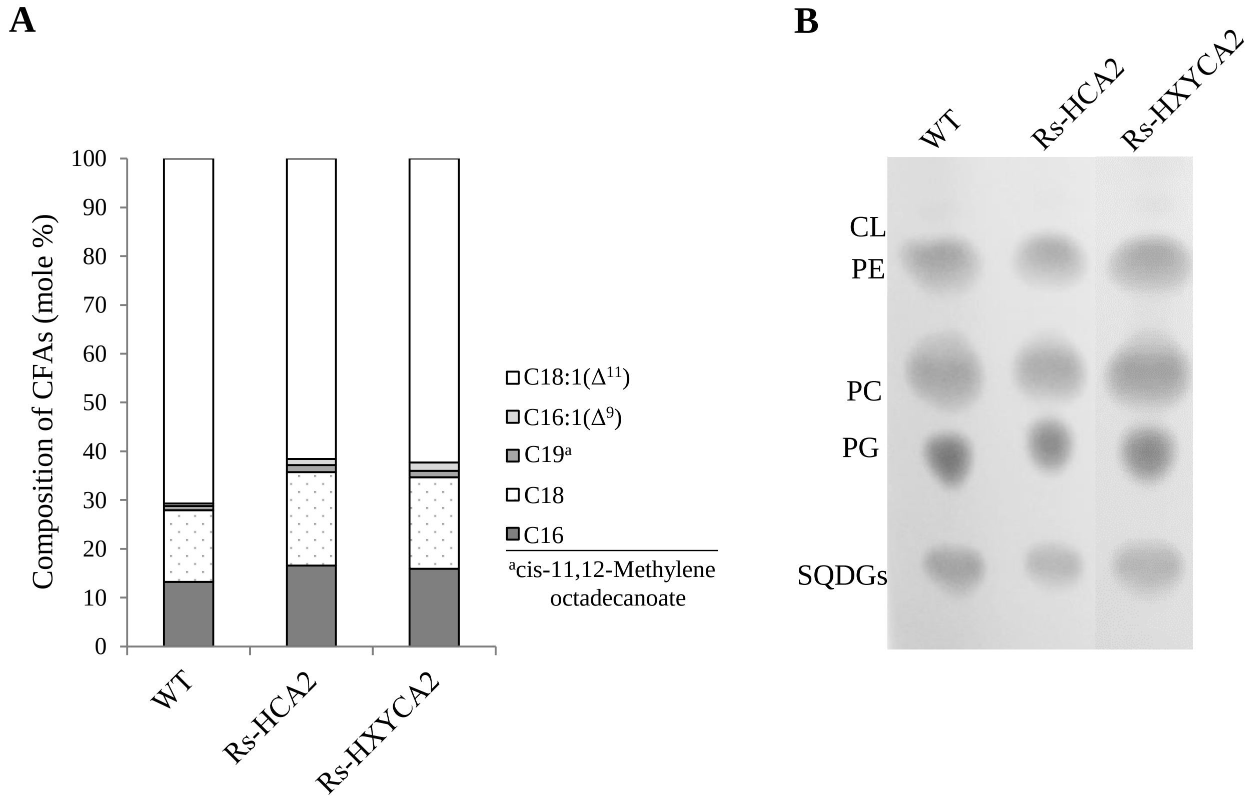

Figure S13. CFA and cellular PL compositions of *R. sphaeroides* WT, Rs-HCA2, and HXYCA2 cells. (A) Cells were grown photoheterotrophically in SisH-D medium and harvested at the exponential growth phase (OD_{660} of 9.5, 5.3 and 5.4 for WT, Rs-HCA2 and Rs-HXYCA2, respectively). CFA compositions were determined from three independent experiments; results were reproduced within SDs of 10-15%, and the data shown are for one of three representative experiments. (B) Membrane lipids of cells grown and harvested as described in (A) were prepared, and aliquots (30 μg each) were analyzed by TLC. All the results shown here were similarly reproduced in three independent experiments, and the data shown are for one of three representative experiments. CL: cardiolipin, PE: phosphatidylethanolamine, PC: phosphatidylcholine, PG: phosphatidylglycerol, SQDGs: sulfoquinovosyl diacylglycerols.

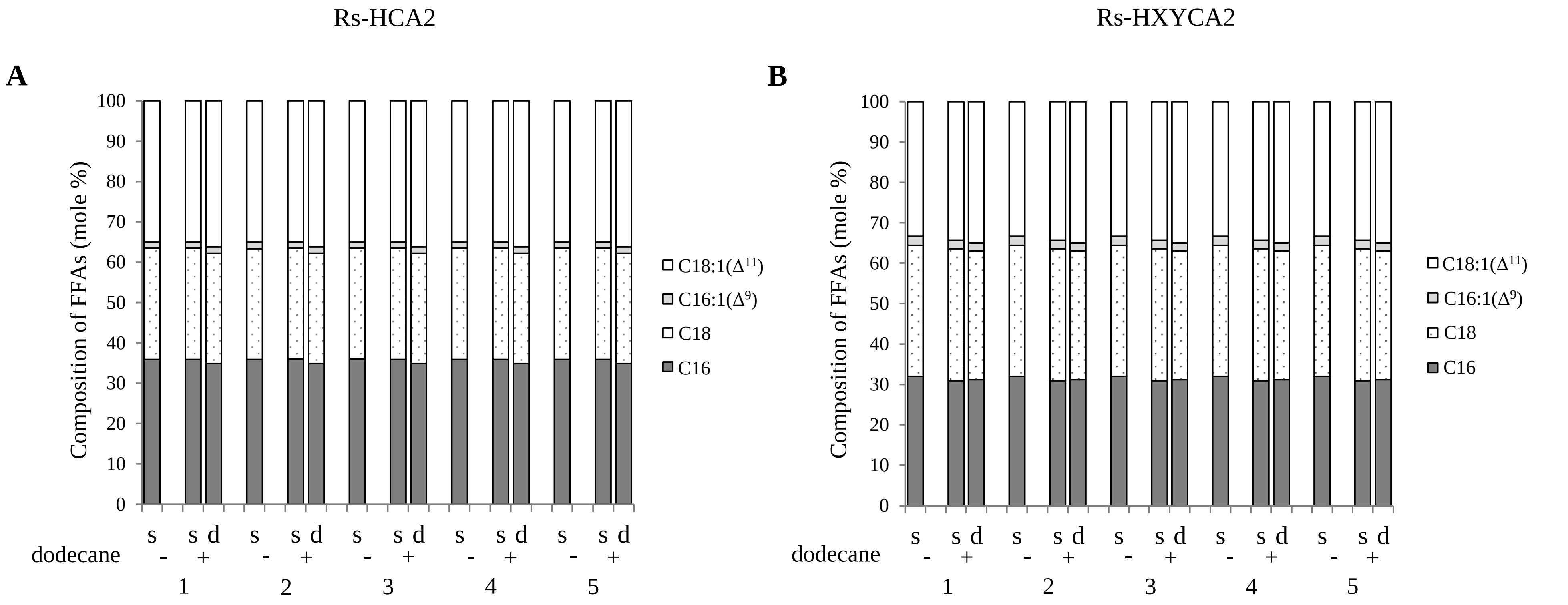

Figure S14. FFA compositions of Rs-HCA2 and Rs-HXYCA2 grown under photoheterotrophic conditions. Rs-HCA2 (A) and Rs-HXYCA2 (B) were grown photoheterotrophically in SisH-D medium with (+) or without (-) 30% dodecane (v/v), and culture aliquots were harvested during growth. The FFAs in the culture supernatant (s) and dodecane layer (d) were determined from three independent experiments. The results were reproduced within SDs of 10-15%, and the data shown are for one of three representative experiments. The numbers 1 to 5 correspond to the time points at which the culture aliquots (and the dodecane in dodecane-overlaid culture) were harvested in Figure 8.

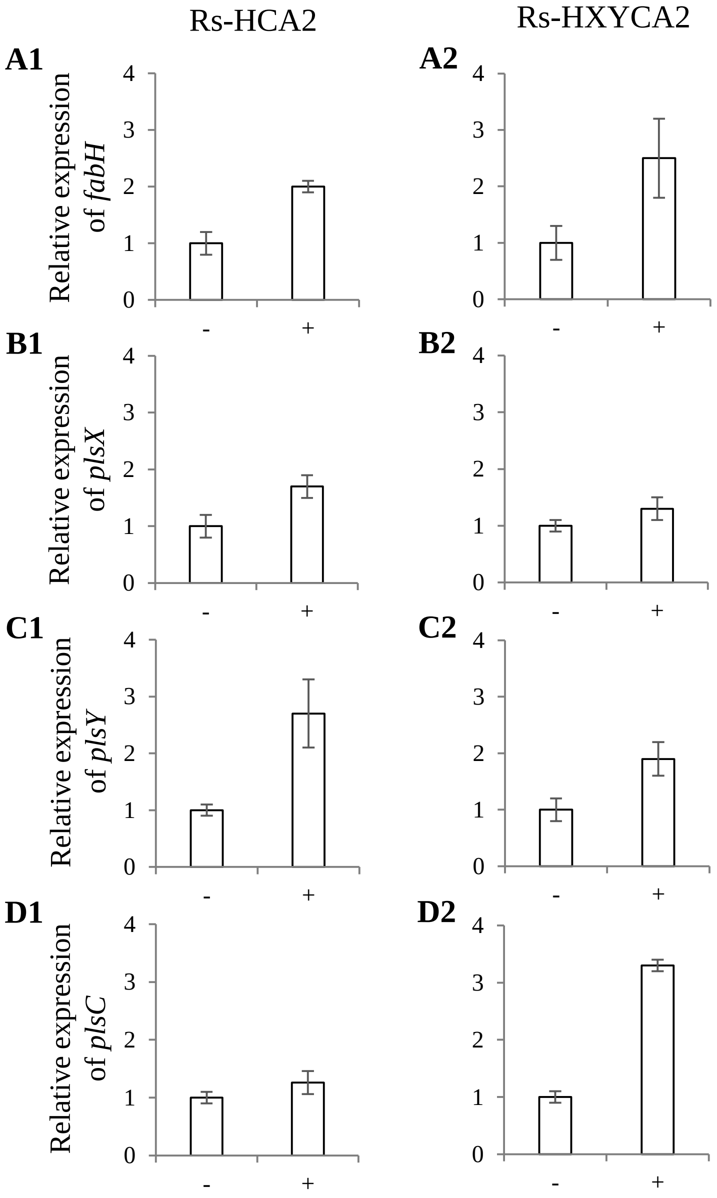

Figure S15. Relative expressions of *fabH*, *plsX*, *plsY*, and *plsC* of Rs-HCA2 and Rs-HXYCA2. Rs-HCA2 and Rs-HXYCA2 were grown photoheterotrophically in SisH-D medium with (+) or without (-) dodecane (30%, v/v), and cells were harvested at the exponential growth phase (OD_{660} of 5.3 and 5.4 for Rs-HCA2 and Rs-HXYCA2 in the absence of dodecane, respectively; OD_{660} of 16.0 and 14.0 for Rs-HCA2 and Rs-HXYCA2 in the presence of dodecane, respectively), Expression levels of *fabH*, *plsX*, *plsY*, and *plsC* were examined by RT-qPCR, and the results in the presence (+) of dodecane were expressed as a mean ratio (± SD) with respect to the corresponding values in the absence (-) of dodecane from three independent experiments. The 16S rRNA was used as a reference gene.

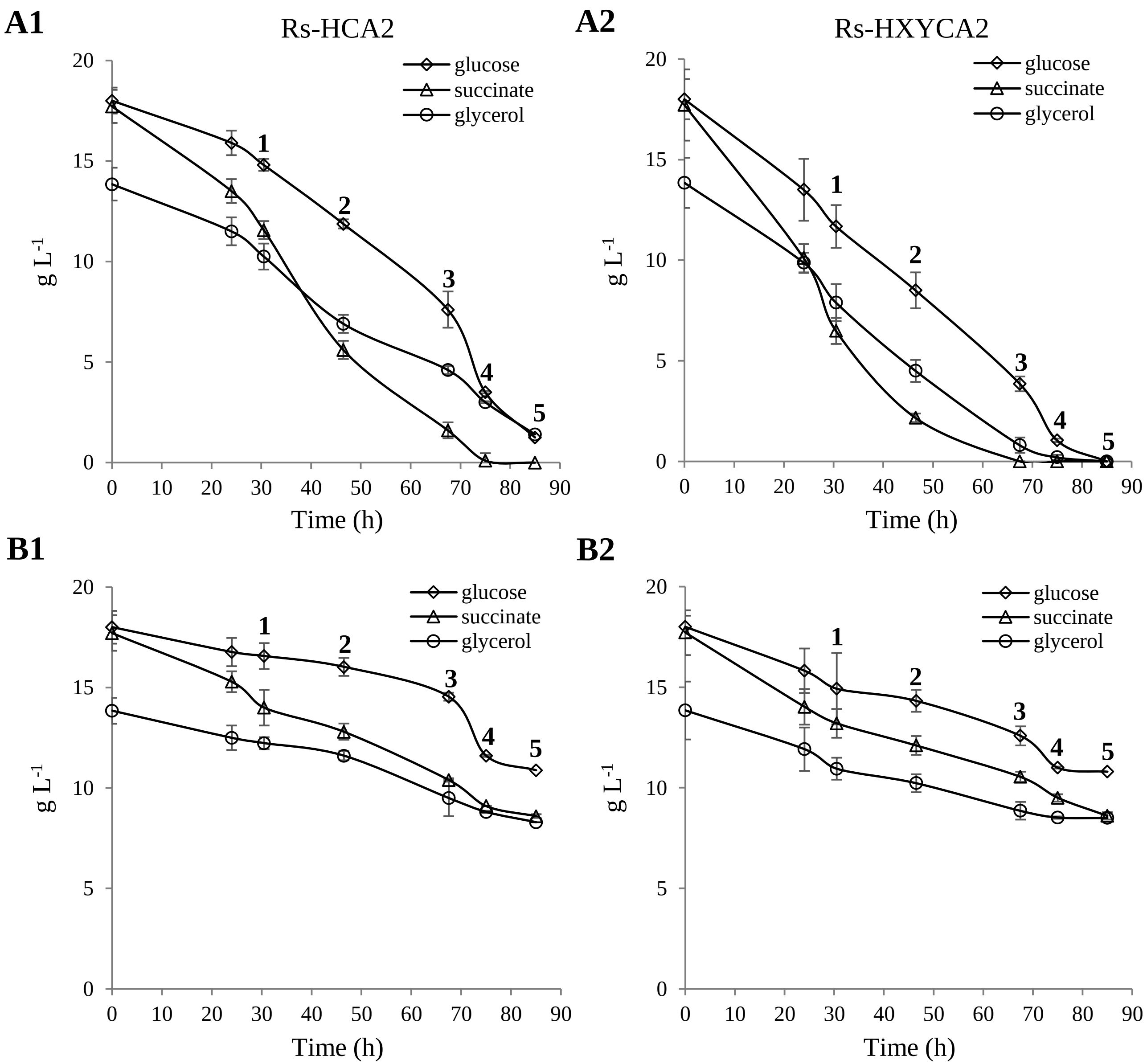

Figure S16. Consumption of carbon sources by Rs-HCA2 and Rs-HXYCA2 grown under photoheterotrophic conditions. (A1, B1) Rs-HCA2 and (A2, B2) Rs-HXYCA2 were grown photoheterotrophically in SisH-D medium with (A1, A2) or without (B1, B2) 30% dodecane (v/v), and cells were harvested during growth. Carbon sources (glucose [diamond], succinate [triangle], and glycerol [circle]) in the culture supernatant were measured and are illustrated with error bars from three independent experiments. The numbers 1 to 5 correspond to the time points at which cells were harvested in Figure 8.
